# Pet and Stray Dogs' Contribution to Zoonotic Transmission Pathways: A Bibliometric Review

**DOI:** 10.1155/tbed/5522451

**Published:** 2025-08-25

**Authors:** Thibaut Langlois, Sylvie Hurtrez-Boussès, Michel de Garine-Wichatitsky

**Affiliations:** ^1^ASTRE, CIRAD, INRAE, University of Montpellier, Montpellier, France; ^2^MIVEGEC, IRD, CNRS, University of Montpellier, Montpellier, France; ^3^Department of Biology-Ecology, Faculty of Sciences, University of Montpellier, Montpellier, France

**Keywords:** bibliometric analysis, canine ecotypes, one health, transmission pathways, zoonotic pathogens

## Abstract

Based on a large-scale bibliometric dataset, domestic dogs (*Canis lupus familiaris*) emerge as the most frequently cited host species in the context of zoonoses, being mentioned in at least 10% of publications for nearly a quarter of the pathogens recognized as zoonotic to humans. This review examines the contributions of pet and stray dogs to various zoonotic transmission pathways, highlighting some mismatches between research focus and actual epidemiological risks. Among zoonotic agents associated with dogs, helminths are disproportionately represented in the literature compared to bacteria and viruses. Pet and stray dogs exhibit distinct zoonotic risks due to differences in exposure patterns and human interactions. Stray dogs are frequently involved in environmentally transmitted diseases, particularly soil- and water-borne parasites, due to uncontrolled defecation and opportunistic behavior. Conversely, pet dogs pose greater risks for direct transmission, particularly via bites, close contact infections, and antimicrobial-resistant bacteria. From a public health perspective, integrating dogs into One Health surveillance frameworks is crucial. Routine genomic monitoring of stray dogs could allow early detection of emerging zoonoses, while large-scale deworming programs, improved sanitation infrastructures, and responsible pet management would mitigate both environmental and direct transmission risks. Vector-borne zoonoses require differentiated control measures, including antiparasitic treatments for tick- and flea-borne infections and environmental interventions for mosquito- and sandfly-borne pathogens. This review focuses on pet and stray dogs only, due to the lack of consistent definitions and data availability for other canine categories. Future research should refine ecological and behavioral studies and dog–host interaction analyses to better quantify the zoonotic risks associated with each dog ecotype and guide targeted intervention strategies. This approach enables a more precise zoonotic risk stratification and contributes to effective disease prevention at the human–animal–environment interface.

## 1. Introduction

Zoonotic diseases, which arise from the close relationship humans have established with surrounding animal species, represent one of the primary threats to global public health [1, 2]. Domestic animals play a key role in the transmission dynamics of these zoonoses due to their abundance and global distribution. The likelihood of pathogen exchange with wild and environmental reservoirs is enhanced through domesticated species, which have central positions in interspecies transmission networks [3]. Thus, they facilitate the gradual adaptation of infectious agents to a wide range of hosts, including humans, acting as intermediaries in the spread of numerous emerging zoonotic diseases [4–8]. This is particularly true for domestic carnivores, which share 90% of their pathogens with other hosts [9]. *Canis lupus familiaris* harbors the greatest diversity of zoonotic infectious agents among all animal species [10]. In this sense, canine populations generally do not constitute an independent reservoir, in which pathogens can persist in the long term: rather, they are part of a broader maintenance complex [11]. As one of the animals interacting most closely with humans within multi-species maintenance communities, the domestic dog is often a major source of zoonotic infections [12, 13].

Beyond the global abundance of canine populations, the primary reason behind the high potential of dogs for transmitting zoonotic pathogens is the intimate and long-lasting relationship they maintain with humans for thousands of years [14]. As a result of this long-term co-evolution, canine pathogens have gradually adapted to humans [10, 15]. This constant proximity across diverse socio-ecological contexts has created multiple interfaces between dogs and humans [16], providing pathogens with privileged access to various zoonotic transmission pathways through a wide range of direct or indirect interactions. Dog bites, for instance, are a common source of bacterial infections [17, 18], in addition to being responsible for almost all cases of human rabies [19]. “Man's best friend” also play a significant role in the epidemiology of brucellosis [20, 21], leptospirosis [22, 23], and Q fever [24, 25], all diseases transmitted through close contact with the bodily fluids of infected animals. Moreover, dogs are recognized as maintenance hosts for numerous (re)emerging zoonotic vector-borne diseases [26, 27], including ehrlichiosis [28, 29], dirofilariasis [30, 31], and leishmaniasis [32, 33]. Finally, canine populations contribute to the spread of a wide variety of environmentally transmitted parasitic infections [34], most of which being categorized as Neglected Tropical Diseases by the World Health Organization [35], such as cystic echinococcosis [36, 37], foodborne trematodiases [38, 39], and soil-transmitted helminthiasis [40, 41]. The diversity of contamination mechanisms associated with dogs therefore highlights the numerous potential pathways through which canine populations can contribute to the emergence of zoonoses.

Although canine populations are not always able to sustain all these pathogens in the long-term, they can still serve as intermediate link for zoonotic infection, acting as epidemiological bridges [42]. By connecting the maintenance complex to the target host, dogs could then facilitate the spread of infectious agents from wildlife to human populations [43]. Far from being an homogeneous entity, the global dog population encompasses a wide range of socio-ecological profiles and lifestyles, each characterized by specific behaviors, habitat preferences, and interactions with humans and other animal species [38, 43–45], thereby influencing disease transmission mechanisms in varied ways. Excluding wild feral dogs, the spectrum of domestic dogs ranges from 'stray dog' (i.e., dogs roaming freely and exposed to wild or commensal fauna without access to a consistent food source or appropriate veterinary care) to 'pet dog' (i.e., dogs living within human households under controlled conditions regarding their displacements, nutrition, and hygiene), with intermediate profiles in terms of management, such as free-roaming owned dogs or dogs kept in shelters or kennels [46–49]. The zoonotic transmission possibilities at either end of the canine ecotypic spectrum are thus likely to vary in both frequency and transmission mode. For instance, pet dogs may be especially more prone to transmit pathogens directly to their owners through close contacts [50], whereas the free defecation of stray dogs in the environment is believed to facilitate the completion of the life cycle of parasites that can be transmitted to humans via foodborne pathways [51]. Given the diversity and complexity of transmission patterns involving dogs, assessing the overall public health impact attributable to *C. lupus familiaris* appears neither realistic nor appropriate. It would nonetheless be conceivable and relevant to break down the continuous gradient of canine ecotypes to estimate their respective contributions to zoonotic transmission pathways.

In this context, associating socio-ecological profiles of dogs with specific zoonotic risks may support the development of effective prevention strategies. Several studies have thus recommended differentiated management of canine populations by focusing on key individuals to reduce their respective roles in zoonotic cycles [45]. For instance, targeted rabies vaccination in Africa and Asia has significantly reduced the number of human cases, particularly in urban areas where stray dogs play a predominant role in rabies transmission [52, 53]. Equipping companion dogs with insecticide-impregnated deltamethrin collars has led to a notable reduction in human visceral leishmaniasis in rural areas of Brazil [54, 55]. Screening and culling programs for infected dogs in commercial breeding kennels, combined with strict biosecurity measures, have helped to limit zoonotic infections by *Brucella canis* [20, 56]. Similarly to these studies, identifying specific transmission pathways of canine pathogens will enable the implementation of targeted health control measures. The aim of this review is therefore to address the diversity of mechanisms by which infectious agents can spread from dogs, focusing on the zoonotic potential of each specific dog ecotype. By synthesizing the available information from the scientific literature, this study seeks to identify overarching trends in public health risks associated with dogs and provide general recommendations for preventing the emergence of zoonoses through the canine epidemiological bridge.

## 2. Materials and Methods

We first compiled a structured list of zoonotic pathogens potentially transmissible by dogs worldwide. Although taxonomic inconsistencies and evolving classifications complicate this task, the resulting inventory captures a broad and representative set of infectious agents relevant to canine-mediated transmission. For each pathogen, we assessed the zoonotic potential and the degree of canine involvement based on available evidence. This step provided an initial framework for understanding transmission dynamics and identifying areas where the epidemiological role of dogs is poorly documented.

To account for potential bias linked to uneven research attention, we conducted a large-scale lexical analysis of a bibliographic database, using the pathogen as the analytical unit. Pigs were included in this analysis as a reference host species, given their established role in zoonotic transmission, their status as livestock animals—epidemiologically and symbolically distinct from dogs—and their position as the second most frequently cited mammalian species in relation to zoonotic transmissions. This approach offers a standardized, quantitative means of comparing the representation of dogs across various zoonotic cycles.

While bibliometric patterns do not constitute empirical proof of transmission, they reveal how scientific discourse reflects—or overlooks—canine involvement in zoonoses. To refine interpretation, we triangulated these results with targeted literature reviews from specialized sources. This integrative method provides a multi-layered perspective on the zoonotic role of dogs, highlighting imbalances in research coverage and supporting the identification of context-specific priorities for surveillance and disease control.

### 2.1. Representative List of Zoonotic Pathogens Transmitted by Dogs

To assess the role of the canine species in various zoonotic transmission pathways, we seek to accurately characterize the contamination mechanisms specific to each pathogen linked to dogs. A list of 335 known zoonotic pathogens, classified by taxonomic groups, was then compiled based on various sources, including Taylor et al. [57], Acha et al. [58], Polack et al. [59], Bauerfeind et al. [60], Rahman et al. [1], the MSD Veterinary Manual [61], and the Centers for Disease Control and Prevention [62]. From these references, key information was extracted for each listed infectious agent regarding the public health impact (low, moderate, high, or priority), the primary source of human exposure (animal, environment, food, or vector) and the main zoonotic transmission pathway (accidental ingestion of plants or insects, consumption of animal products, inoculation via bites or scratches, physical or close contact, transmission by ectoparasites, transmission by flying arthropod vectors, consumption of fish or seafood, inhalation, transmission through contact with soil, waterborne transmission, or unsanitary conditions route).

Additional bibliographic searches were then conducted using the Google Scholar database to highlight recognized or strongly suspected transmission cases originating from dogs. If the ability of *C. lupus familiaris* to transmit the infectious agent to humans was confirmed, the next step would be to estimate, through targeted bibliographic research, whether dogs primarily act as the primary source of zoonotic infection (main transmission host), as a secondary source (frequently implicated host), as an occasional source (a few confirmed cases), or as a potential source (competence demonstrated only in laboratory settings). Eco-epidemiological studies specifically exploring the roles of different types of dogs in the transmission of certain listed pathogens have also been referenced in Supporting Information 1: Table S1, where all the bibliographic information compiled with the corresponding sources is presented. To further investigate the diversity of pathogens transmitted to humans by dogs and the factors that determine this canine competency, *χ*^2^ tests were performed to identify potential significant associations. Adjusted standardized residuals were calculated to assess the extent to which the proportion of zoonotic pathogens transmitted by dogs differed by transmission pathways (Supporting Information 2), taxonomic groups and health impact. Data processing, statistical analyses, and graphical representations were performed using R software (version 4.3.1), using the “dplyr,” “tidyr,” “ggplot2,” and “gt” packages (Zoonotic list analysis code available in the Supporting Information 2).

### 2.2. Bibliometric Analysis to Assess the Pathogen/Dog Association

**Figure 1:**
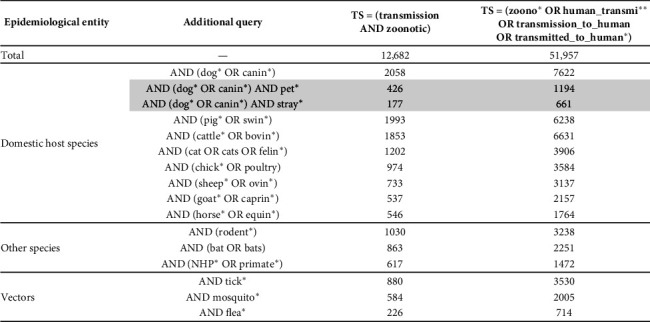
Number of publications provided per query in the Web of Science database.

To evaluate numerically the overall zoonotic involvement of *C. lupus familiaris* based on the scientific literature, we aim to determine the extent to which canine ecotypes are associated with each listed pathogen at the scale of a bibliographic database. A bibliometric analysis was then conducted on a macroscopic scale using the Web of Science (WoS) Core Collection database as of November 15, 2024. First, we compared the number of results from a search for “dog(s)” in relation to “zoonotic transmission” to different queries searching for other host species or reservoirs of zoonotic importance (number of results for each query compiled in Figure 1). For each of these 335 zoonotic infectious agents on the list, the number of English-language Articles or Reviews mentioning the scientific name of the pathogen (or its abbreviation with the genus shortened) in their topics (TS = titles, keywords, or abstracts) was recorded in column “Total” in Supporting Information 1: Table S1. Among these publications, those that also mentioned the words “dog(s)” and “pig(s)” were counted in the “With_dogs” and “With_pigs” columns, and those that cited the words “pet(s)” and “stray,” in addition to “dog(s),” were counted in the “With_pet_dogs” and “With_stray_dogs” columns, respectively. The searches conducted using terms referring to shelter or free-roaming dogs did not yield enough results to be accounted for. The queries entered in the Web of Science database are available in Supporting Information 1: Table S1. Example to complete the “With_pet_dogs” column: TS = (*Microsporum_canis* AND dog*⁣*^*∗*^ AND pet*⁣*^*∗*^) AND (LA = English) AND (DT = (Article OR Review)). The factors “Dog's part” ( = “with_dogs”/“total”), “Pig's part” ( = “with_pigs”/“total”), “DogPig's part” ( = log (“with_dogs” + 1) – log (“with_pigs” + 1)), and “Dog's partition” ( = log (“with_stray_dogs” + 1) – log (“with_pet_dogs” + 1)) were then calculated to classify pathogens based on their association with dogs (or pigs).  Table 1 includes only pathogens mentioned in more than 100 publications (Total > 100) for which the dog is considered competent (or suspected to be). For the full list of documented zoonotic pathogens, missing information—including WOS queries, number of publications, reservoirs, geographical context, and canine competence—as well as the corresponding bibliographic sources, is available in the full version of Supporting Information 1: Table S1.

A first logistic binomial regression model was selected to evaluate the effects of pathogens' “taxonomic groups”, “transmission pathways” and “health impact” on the probability of mentioning dogs in scientific publications, considering articles “with dogs” as the number of successes out of a “total” number of trials. As a potential epidemiological bridge host frequently involved in zoonotic transmissions [63], the pig was then selected as a reference control species to compare the overall involvement of the dog in the transmission of recognized pathogens to humans. A second logistic binomial regression model was then used to examine the effect of the same explanatory variables on the probability of citing “dog(s)” (coded as success) compared to “pig(s)” (coded as failure). This choice was further supported by bibliometric data, as pigs were the second most frequently cited mammalian species in the Web of Science database in the context of zoonotic transmission. The models including the interaction, selected using Akaike's Information Criterion (AIC), have a residual deviance of 47,403 and 21,316, respectively (with 245 and 229° of freedom) versus a null deviance of 137,487 (Model 1) and 61,623 (Model 2), indicating a substantial fit to the data. For these two statistical models, only the 310 pathogens cited in at least 50 publications were retained to minimize biases caused by small sample sizes. Additionally, 16 extra pathogens, for which dogs and pigs were cited in fewer than 5 articles in total, were excluded from Model 2.

**Figure 2:**
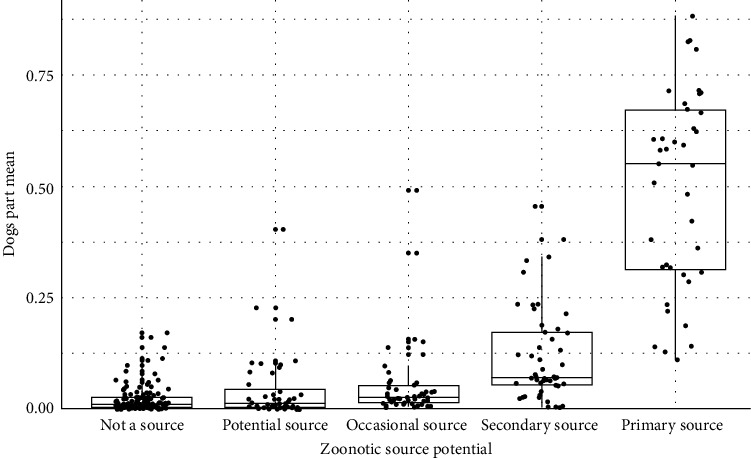
Distribution of “Dog's part” mean by zoonotic potential of dogs.

Finally, a third logistic binomial regression model was used to examine the effect of the same explanatory variables on the probability of citing stray dogs (as a success) compared to pet dogs (as a failure). This time, only the 141 pathogens mentioned in at least 5 publications, citing either “pet dogs” and/or “stray dogs,” were retained for this last model, which fitted the data accurately after AIC minimum selection (Model 3 residual deviance of 403 with 85 df against 1406 initially). Using the “dplyr,” “emmeans”, “gt,” and “ggplot2” packages, model summary tables and predicted probability plots were created on R software (version 4.3.1) for the three models (Bibliometric analysis code and Model summaries available in the Supporting Information 2). To perform a cross-validation of bibliographic and bibliometric data, a Spearman correlation test was conducted to assess the relationship between the ordinal variable “Canine zoonotic potential” and the “Dog's part” index, whose distribution is shown in Figure 2.

## 3. Results

### 3.1. Representative List of Zoonotic Pathogens Transmitted by Dogs

**Figure 3:**
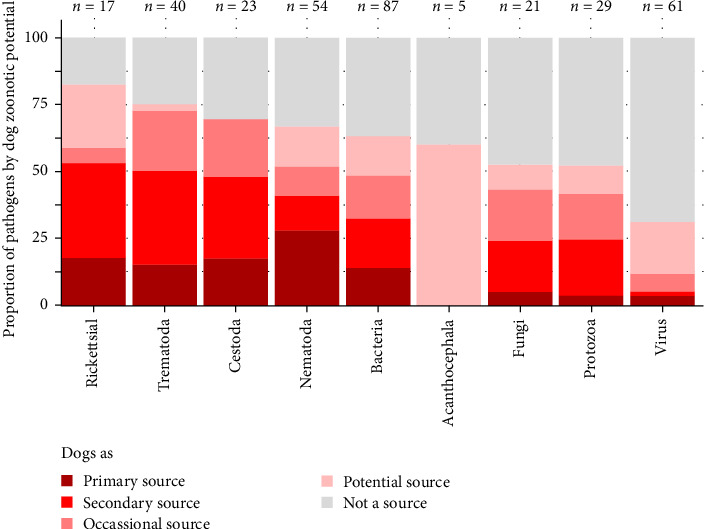
Number of zoonotic pathogens by taxonomic groups.

**Figure 4:**
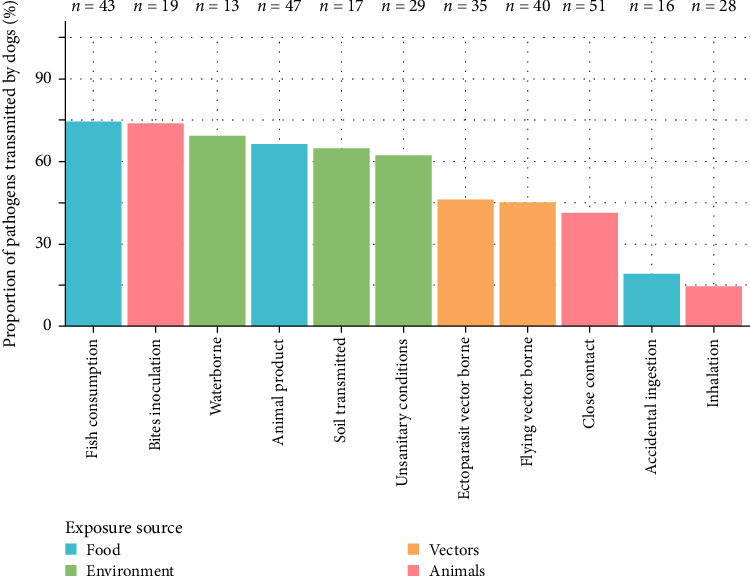
Number of zoonotic pathogens by transmission pathways.

Literature searches revealed 152 pathogens that *C. lupus familiaris* is able to transmit to humans, representing almost half of the zoonotic agents listed in our study. Dogs play the role of primary host for human transmission for 44 of them, while they may act as a secondary zoonotic source for 60 pathogens and are occasionally involved in the transmission of the remaining 48. To our knowledge, no documented cases of zoonotic transmission by dogs have been reported under natural conditions for the remaining 185 listed pathogens, although 117 of them appear capable of infecting dogs. Among these, dogs may pose a potential zoonotic risk for an additional 46 pathogens, as canine competence remains neither confirmed nor ruled out due to conflicting sources or studies demonstrating transmission potential only under laboratory conditions. Figures 3 and 4 show the proportion of zoonotic pathogens transmitted by dogs across the list, by taxonomic groups and by routes of transmission, respectively.

Figure 3 highlights some taxonomic groups with a higher proportion of zoonotic pathogens transmitted by dogs than others (*χ*^2^*p*-value = 2.558e–04), particularly helminths (adjusted *χ*^2^ residuals = 1.98). Conversely, the proportion of viruses for which dogs are competent hosts is significantly lower than expected (adjusted *χ*^2^ residuals = −2.82).  Figure 4 shows that the proportion of zoonotic pathogens transmitted by dogs varies according to the mode of transmission of infectious agents (*χ*^2^*p*-value = 1.156e–06). Dogs are particularly competent for most pathogens transmitted through food consumption (especially fish or seafood: adjusted *χ*^2^ residuals = 1.99), whereas they are generally not competent for those transmitted through direct contact with animals, except in the case of bite inoculations (adjusted *χ*^2^ residuals = 1.28). See details of the results of *χ*^2^ test by zoonotic potential of dogs in the Supporting Information 2 (*χ*^2^ statistic = 97.4; df = 40; *p*-value = 1.098e–06).

### 3.2. Bibliometric Analysis to Assess the Pathogen/Dog Association

Figure 1 presents the number of publications citing domesticated host species, major wild reservoirs, and arthropod vectors. It shows that dogs are the most cited animal compartment in the context of zoonotic transmissions, in more than one in seven publications, ahead of pigs, cattle and cats. Pigs, reported at levels comparable to those of dogs, will later be used as a control for comparison with livestock species.

The bibliometric analysis conducted on the WoS Core Collection database measured the proportion of publications mentioning the term “dog” for each of the listed pathogens, thereby identifying the zoonotic agents most associated with *C. lupus familiaris* in the scientific literature. At the top of the ranked list, as shown in Table 1, are *Helicobacter canis*, *Trichuris vulpis*, *Ehrlichia canis*, *Capnocytophaga canimorsus*, *Staphylococcus pseudintermedius*, *B. canis*, *Dipylidium caninum*, *Uncinaria stenocephala*, *Bartonella vinsonii berkhoffii*, and *Anaplasma platys*. In addition to this top 10, dogs are mentioned in at least one out of every two publications (Dog's Part Index > 0.5) for 15 other pathogens, compared to only three pathogens for pigs. Among the pathogens cited in at least 50 publications, 77 had a Dog's Part Index exceeding 0.1, whereas pigs were mentioned in more than one in 10 articles for only 42 listed pathogens. Comparatively, the dog index is twice as high as the pig index when averaged across all listed zoonotic pathogens (mean Dog's part = 0.105; mean Pig's part = 0.053). Most pathogens listed have more mentions of pigs than dogs (DogPig_part's > 0 for 139 zoonotic agents compared to 165). However, dogs are cited at least 10 times more than pigs for 56 pathogens, while pigs are cited 10 times more than dogs for half as many (DogPig_part's < 1 for 24 pathogens). Compared to pigs, these results suggest that dogs hold a relatively important place in the epidemiological dynamics of the pathogens they are associated with.

As a cross-validation of the classification established in the previous section, the Spearman's correlation test indicates a very significant positive relationship between “Dogs part” index and Zoonotic potential of dogs (*p*-value < 2.2 × 10⁻^16^; rho = 0.668). Thus, we observe in Figure 2 that pathogens for which dogs serve as the primary source of zoonotic infections have a relatively high Dog's part mean of 0.495, although the index exhibits considerable variability (standard deviation (sd) = 0.222). This value progressively decreases depending on the role of dogs in pathogen transmission: 0.117 when dogs act as secondary transmission hosts (sd = 0.105), 0.056 for pathogens that dogs occasionally transmit to humans (sd = 0.089), 0.044 for potential but unconfirmed zoonotic sources (sd = 0.076), and finally, 0.022 for pathogens that dogs do not transmit to humans (sd = 0.031).

Binomial logistic regression Models 1 and 2 demonstrated a significant effect (*p*-value < 2.2 × 10⁻^16^) of taxonomic groups, transmission routes, health impact, and their interactions, on the probability of mentioning dogs, respectively, on total publications or compared to pigs. Dogs were three times more likely to be cited in publications referencing pathogens with low (*p*=0.059) or moderate (*p*=0.043) public health impacts than in those concerning pathogens of high (*p*=0.015) or priority (*p*=0.016) importance. It is worth noting that this trend is not as pronounced for pigs, even though Model 2 does not indicate significant differences between the two host species in terms of probabilities. Additionally, rickettsial organisms, cestodes, and nematodes were frequently associated with dogs in the scientific literature, exhibiting significantly higher predicted probabilities of mentions (*p*=0.183, *p*=0.124, and *p*=0.098, respectively in Model 1) compared to other pathogen groups, notably viruses (*p*=0.011) or bacteria (*p*=0.019). Comparatively, rickettsial species, protozoa, and helminths are more likely to be cited alongside dogs than pigs (*p*  > 0.5 in Model 2).

**Figure 5:**
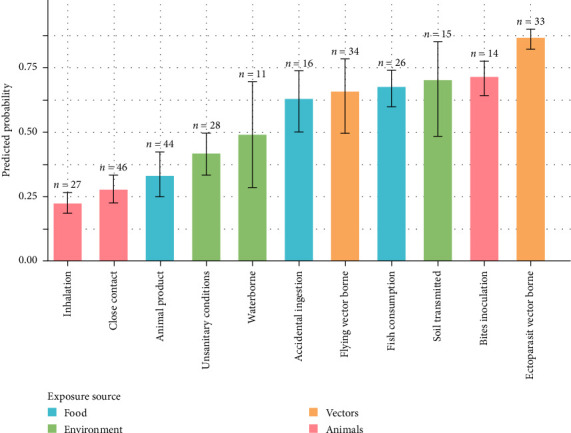
Probability of citing “dogs” against “pigs” by transmission pathways.

The results of the Model 1 indicate that pathogens transmitted through bites (*p*=0.150), via ectoparasites (*p*=0.084) or contact with soil (*p*=0.078), are associated with the highest frequencies of dog mentions (Figure and summary in Supporting Information 2). Moreover, Model 2 shows that dogs are significantly more frequently cited than pigs (*p*  > 0.5) for these three transmission modes, as well as for fish or seafood consumption, flying vector borne and accidental ingestion (Figure 5). Conversely, pathogens transmitted through inhalation (*p*=0.009 in Model 1) and close contact (*p*=0.0015 in Model 1), as well as those associated with animal products and poor hygiene, are more frequently linked to pigs (*p*  < 0.5 in Model 2, Figure 5). This suggests that dogs are less commonly cited in these contexts than livestock species. On a broader scale, the frequency of dog citations varies significantly depending on the exposure source considered. Dogs are most frequently mentioned in cases of vector-borne transmission (*p*=0.042 [0.028; 0.062]), followed by foodborne transmission (*p*=0.033 [0.027; 0.041]), environmental transmission (*p*=0.027 [0.018; 0.038]), and finally, direct transmission (*p*=0.021 [0.017; 0.025]). Model 2 indeed indicates that only vectorial exposure is more strongly associated with dogs than with pigs (*p*=0.744), whereas environmental and animal exposure sources are primarily linked to pigs (*p*  < 0.5), as can be seen in Figure 5, which compares the “dog” and “pig” citations by transmission pathways.

In a second step, by comparing the number of publications citing the terms “stray dogs” and “pet dogs,” we aim to assess the extent to which each zoonotic pathogen is associated with one or the other canine lifestyle in the scientific literature. The 10 infectious agents with the highest *Dog's Partition Index*, and thus most frequently associated with stray dogs compared to pet dogs, are as follows: *Heterophyes* spp., *Dioctophyme renale*, *Toxascaris leonina*, *D. caninum*, *Ancylostoma braziliense*, *Leptospira interrogans Copenhageni*, *H. canis*, *Echinococcus granulosus*, *Dibothriocephalus latus*, *and Macracanthorhynchus* spp. Conversely, the 10 pathogens most frequently associated with pet dogs compared to stray dogs are: *Staphylococcus intermedius*, *Clostridioides difficile*, *Staphylococcus aureus*, *Streptococcus canis*, *C. canimorsus*, *S. pseudintermedius*, *Salmonella typhimurium*, *Bordetella bronchiseptica*, *Clostridium perfringens*, *and Yersinia enterocolitica*.

The results of the binomial logistic regression Model 3 assessing the probability of citing “stray dogs” compared to “pet dogs” in publications mentioning pathogens transmitted by dogs are summarized in the Supporting Information 2 across different taxonomic groups, health impacts, and transmission pathways. The deviance analysis indicated significant contributions of the three factors, especially Pathway and Taxonomic group, and their interaction (*p*-value < 2.2 × 10^−16^). Zoonoses caused by cestodes (*p*=0.557), trematodes (*p*=0.465), nematodes (*p*=0.428), rickettsiae (*p*=0409), and protozoa (*p*=0347) appear to be more frequently associated with stray dogs in the literature, whereas bacteria (*p*=0.175), fungi (*p*=0.127), and viruses (*p*=0.005) are significantly linked to companion dogs. Additionally, no significant differences were found in the predicted probabilities across different levels of public health impact, although companion dogs appear to be associated with more concerning pathogens than stray dogs.

**Figure 6:**
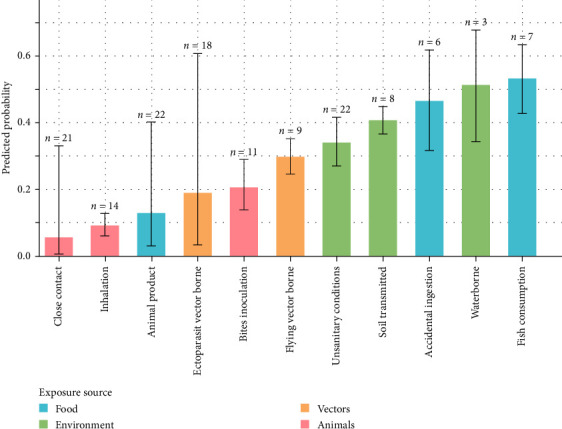
Probability of citing “stray dogs” against “pet dogs” by transmission pathways.

Furthermore, when pathogens are grouped by exposure source, diseases related to direct exposure to “Animals” are significantly less frequently cited in the context of stray dogs compared to other type of exposure (*p*=0.156 [0.119; 0.202]). Specifically, pathogens transmitted through direct interactions with animals, such as inhalation (*p*=0.089), close contact (*p*=0.053) or bite inoculation (*p*=0.204), demonstrated significantly lower predicted probabilities at the 95% confidence level and were therefore more frequently associated with pet dogs (Figure 6). Conversely, publications reporting pathogens transmitted through fish consumption showed the highest predicted probability of mentioning stray dogs compared to pet dogs (*p*=0.533), followed by water contamination (*p*=0.512) and soil-borne pathogens (*p*=0.464).

## 4. Discussion

### 4.1. Functional Roles, Host Status, and Scientific Representations of Dogs in Zoonotic Transmission

This review highlights the epidemiological significance of *C. lupus familiaris* as the most frequently cited host species in the context of animal-to-human transmission. This prominence is primarily explained by the species' ecological versatility and ubiquity in domestic environments, where dogs are involved in a wide range of interspecific transmission pathways [43, 63]. In accordance with Morand et al. [10], the proportion of infectious agents shared between dogs and humans is likely the highest in the animal kingdom, largely reflecting the long evolutionary history shared by both species. Despite this apparent centrality, 20% of documented zoonotic pathogens have never been detected in dogs. Moreover, they often act as zoonotic dead-end hosts for nearly half of the listed pathogens, with little or no confirmed human transmission. Nevertheless, our bibliographic analysis indicates that dogs still play a significant role in the infectious dynamics of more than a 100 zoonotic agents, even when they are not essential to pathogen persistence. Indeed, their role in pathogen maintenance clearly extends beyond the 20 zoonotic agents for which they are identified as primary reservoirs.

Even when they do not serve as core maintenance hosts, dogs can facilitate the circulation of numerous generalist pathogens within multi-host communities. Through their interactions with humans, wildlife, and other domestic animals, they contribute to the formation of maintenance complexes, as described by Haydon et al. [11]. In such systems, dogs often act as relay hosts, spatial vectors, or mechanical carriers, rather than as reservoirs per se. Their pivotal function as an intermediary compartment bridging sylvatic and domestic transmission cycles thus appears to be crucial [64–66]. This intermediary role aligns with the definition of “bridge hosts” proposed by Caron et al. [42], which describes species that sustain continuous epidemiological connections between maintenance reservoirs and final hosts—namely humans. The high density, ecological diversity, and social connectivity of dog populations contribute to this bridging function, enabling sustained spillover potential across ecological and social boundaries.

While lexical counts do not allow for precise estimation of the infectious burden attributable to dogs, bibliometric analysis provides a valuable framework to explore how different transmission pathways are associated with canine populations in the scientific literature. This approach allows for a synthesis of the representations attached to the zoonotic role of dogs and offers interpretative tools to anticipate potential emergence risks. Moreover, it facilitates interspecies comparisons of zoonotic potential, supporting the prioritization of health threats based on their perceived weight and visibility. The comparative analysis between pets and livestock, for instance, lays the groundwork for a functional typology of host species—one based not only on biological traits and lifestyles but also on the scientific representations that shape surveillance strategies. Despite a similar publication volume on zoonotic agents when compared to pigs or cattle, dogs appear to be associated with a broader spectrum of pathogens and transmission mechanisms, suggesting a particularly extensive involvement in infectious dynamics. This overrepresentation is especially marked for certain taxonomic groups, such as cestodes, nematodes, and rickettsiae, and for specific transmission routes. While dogs are often implicated in vector-borne, environmental, and bite-related zoonoses, livestock—primarily represented by pigs—tend to be more frequently involved in foodborne, hygiene-related, and close-contact transmissions, reflecting the exposure contexts typical of farming systems.

### 4.2. Canine Ecotypes and Their Epidemiological Roles in Zoonotic Transmission

The global dog population displays considerable ecological heterogeneity, with individuals variably connected to both pathogen maintenance complexes and human populations, depending on their lifestyle and level of integration [44, 45]. This diversity is rarely reflected in review studies, which often rely on simplified classifications. For methodological consistency, our analysis focused on the two most frequently cited terms in the corpus: *pet dog* and *stray dog*, used as proxies for the two ends of the ecotypic gradient. Other categories commonly employed in field studies—such as *free-roaming*, *community*, *owned*, or *unrestrained*—were too inconsistently cited to support robust comparisons. This limited lexical scope reveals a pronounced polarization in scientific representations of zoonotic risk. Pathogens associated with environmental or parasitic transmission are more frequently linked to stray dogs, often portrayed as diffuse sources of contamination outside human control structures. In contrast, pet dogs are more commonly associated with bacterial infections transmitted through close contact, such as bites or licking. These distinctions help shape contrasting epidemiological profiles: stray dogs are perceived as external, unregulated threats, while pet dogs are seen as intimate companions, potentially facilitating pathogen adaptation within household environments.

Ecological evidence supports this dichotomy. Stray dogs, due to frequent exposure to contaminated environments and the lack of routine veterinary care, are prone to accumulating and disseminating resilient pathogens through their interactions with multi-host systems [51, 67]. Conversely, companion dogs, although generally less exposed to environmental reservoirs, remain deeply embedded in human social spaces. Their proximity to humans creates repeated opportunities for direct transmission and may promote the gradual adaptation of pathogens to human hosts [14, 68, 69]. As the final interface before human infection, the role of pet dogs as potential zoonotic amplifiers should not be underestimated. These findings highlight the importance of integrating ecotype-specific characteristics into zoonotic risk assessment. From a public health perspective, tailored interventions are needed to address the distinct functions of each ecotype within transmission networks. Stray dogs could be prioritized in deworming campaigns aimed at reducing environmental parasitic loads, while in contexts of viral emergence, restrictions on close human–pet interactions may be necessary. More broadly, acknowledging the functional diversity of dogs is essential to developing health management strategies within an ecosystemic framework.

Bibliometric analyses remain heavily dependent on the explicit use of keywords such as *dog*, *stray*, and *pet*, which may reflect editorial conventions rather than precise epidemiological distinctions. The lack of standardized terminology for intermediate profiles makes them difficult to identify automatically and restricts analysis to a binary model. While such lexical simplification is operationally useful, it hampers the classification of ecotypes and limits the ability to accurately represent the complexity of canine populations in real-world contexts, particularly in rural and peri-urban settings, where ownership, mobility, and control are often ambiguous. Despite being underrepresented in the literature, intermediate canine profiles such as free-roaming owned dogs likely play a key epidemiological role. These dogs are omnipresent across rural, peri-urban, and urban environments and often maintain interactions with both confined pets and unregulated stray populations. Acting as epidemiological connectors, they may facilitate the movement of pathogens across ecological and social boundaries [38, 44, 70, 71]. As such, interconnected and sympatric canine subpopulations could contribute to each stage of the species barrier crossing [72], progressively bringing wildlife-origin pathogens closer to human settlements [38, 43] and amplifying the risk of spillover.

### 4.3. Fecal Contamination and Environmental Transmission

Dogs frequently serve as definitive hosts for a wide diversity of helminth species, shedding infective stages (e.g., eggs or larvae) into the environment [51, 59], where they can persist for extended periods and further infect humans through soil, water, or food contamination [34, 73]. These parasites have prolonged life cycles and exceptional environmental resilience. This allows them to persist in human habitats, facilitates continuous transmission, even in the absence of direct host-to-host contact [74]. For instance, *Toxocara canis*, *Trichuris vulpis*, and several Ancylostomatidae—nematode species highly specialized for canine hosts—can survive in the soil for months to years, creating long-lasting sources of exposure [75, 76].

Moreover, stray dogs are more frequently mentioned in the literature for environmentally transmitted helminths, which supports their role in maintaining resistant parasites through open defecation in domestic habitats, public spaces and cultivated agricultural areas [77–81]. The risks of contamination of water sources, other domestic animals and crops, then appear to be particularly high. Beyond passive fecal–oral transmission, stray dog populations actively contribute to the completion of heteroxenous parasitic cycles through their predatory and opportunistic behavior towards intermediate hosts (e.g., aquatic prey, rodents, and livestock carcasses) [38]. The spread of pathogens such as *Echinococcus* spp. and Fish-borne Zoonotic Trematodes [82–84] is therefore facilitated. In the absence of deworming treatments, roaming dogs, which are more frequently exposed to contaminated environments, could then serve as key local reservoirs, supporting the transmission of neglected tropical diseases by significantly increasing the parasitic load in favorable wetland ecosystems [51, 67, 85].

To mitigate the role of stray and free-roaming dogs in the persistence and transmission of environmental parasites, targeted control strategies must be implemented. Large-scale deworming campaigns should be prioritized for these high-risk canine populations to reduce the environmental burden of helminths and limit parasite dissemination [86]. Improving sanitation infrastructures, including proper waste disposal and the management of dog feces in urban and peri-urban areas, appears to be essential to curbing soil and water contamination [78, 87]. Finally, community awareness programs can encourage responsible pet ownership by promoting movement control and appropriate veterinary monitoring and avoiding abandonment [88, 89].

### 4.4. Direct Exposure to Animals and Close Contact Transmission

Our bibliographic research indicates that dogs are highly competent hosts for most zoonotic pathogens transmitted via bites or scratches. Dogs indeed play a major epidemiological role in the transmission of rabies [90], as well as in numerous bacterial superinfections resulting from bite wounds (*Capnocytophaga* spp., *Staphylococcus* spp., *Pasteurella* spp., and *Streptococcus* spp.) [17, 18]. As a result, they are frequently mentioned in scientific publications focusing on these biological agents, particularly in comparison with domesticated artiodactyls, such as pigs, cattle, and small ruminants.

However, the overall epidemiological involvement of dogs in other forms of direct animal-to-human transmission seems limited. Apart from a few canine-adapted bacteria with which they have co-evolved, such as *H. canis*, *B. canis*, and *Corynebacterium auriscanis*, dogs rarely act as efficient vectors for airborne or close-contact zoonotic infections. This explains their overall weak association with viruses (and, to a lesser extent, bacteria, and fungi) in the scientific literature, in accordance with the data presented by Olival et al. [91] and Han et al. [92]. Indeed, pathogens with low environmental persistence generally rely on frequent host-to-host interactions for transmission [93, 94]. For this reason, their favored hosts are species that form dense populations in the wild, such as bats [95], or those raised in large numbers in intensive farming systems [96]—two conditions that typically do not apply to canine populations. While dogs remain competent hosts for many of these pathogens, they are less frequently implicated in epidemiological cycles requiring high infectious pressure for zoonotic transmission. In contexts of poor hygiene conditions or contamination of animal products, the role of dogs appears secondary to that of the livestock species, as represented by pigs in our study. This is particularly the case for bacteria with broad host spectra (e.g., *Salmonella* spp., *Clostridium* spp., *Campylobacter* spp., EHEC, and antibiotic-resistant strains), whose transmission is primarily facilitated in high-density farming environments [97]. Comparative studies should thus refine species-specific intervention strategies according to the associated risks, such as deworming programs for dogs and biosecurity measures for zoonotic bacteria in livestock farming.

Viruses and bacteria are also significantly less frequently reported in association with stray dogs, whereas companion dogs appear more often in publications related to airborne pathogens, close contact infections, and bites. Enclosed settings and the intimate pet/owner interactions could then promote prolonged and repeated exposure to animals, facilitating the sustained spread of directly communicable pathogens [98–100]. In contrast, while stray dogs may harbor many zoonotic pathogens, they interact less frequently and closely with humans, which reduces direct transmission risks [51]. This pattern supports the broader observation that zoonotic diseases requiring direct contact are more prevalent in domestic environments where livestock and pets represent an increased risk. Meanwhile, stray animals are more frequently associated with environmentally mediated infections due to their higher exposure to persistent sources of contamination.

In high-risk settings, health awareness programs contribute to educating pet owners on bite prevention and wound management [101]. In order to mitigate the risks of canine-origin zoonotic transmission through direct contact, routine veterinary monitoring of companion animals must be put in place, including regular health check-ups [68, 69]. A rational management of antimicrobials administered to dogs will also help preventing the development of antibiotic-resistant bacterial strains [102]. In kennels and shelters, strict hygiene protocols, including disinfection, isolation of symptomatic animals, proper ventilation, and routine veterinary care, are essential to limiting pathogen transmission and ensuring animal health [103].

### 4.5. Vector-Borne Transmission Pathways

Although dogs appear to transmit only a small proportion of all vector-borne pathogens, they are frequently cited in the scientific literature [27, 104, 105], suggesting a high degree of host specificity for many of these zoonotic agents. This specialization imply that dogs often play a crucial role in the vector-borne transmission cycles in which they are involved, especially for rickettsia species that are well adapted to their main host (e.g., *Anaplasma platys* or *E. canis*) [29, 106]. This is indeed particularly true for pathogens transmitted by ticks or fleas, as dogs are significantly less frequently associated with pathogens carried by flying blood-feeding insects. This distinction likely reflects the feeding preferences and ecological niches of different arthropod vectors [107, 108]. Although canine populations contribute to the maintenance of pathogens transmitted by mosquitoes, sandflies, and other biting flies, these vectors generally transmit infections to humans without direct involvement of dogs, as observed with *Brugia* spp. [109] and *Leishmania* spp. [110]. Indeed, flying insects opportunistically feed on various mammals, a pattern confirmed by comparisons with pigs, where both species serve at equivalent levels.

No significant differences are also observed at a finer scale between canine ecotypes regarding flying vector-borne pathogens. In contrast, transmission via fleas and ticks appears to be particularly specific to dogs compared to pigs, with pets cited more often than stray dogs in transmission cycles involving ectoparasites. Although this last distinction is not statistically significant, it suggests that companion dogs may play an essential role in transporting and sustaining infected vectors within human households [111, 112]. To mitigate vector-borne zoonotic risks associated with companion dogs, regular antiparasitic treatments, including acaricides and insecticidal collars, are crucial, particularly in endemic areas, to curb the proliferation of ticks and fleas within human settings [113, 114].

Although bibliometric analyses generally align with the actual zoonotic potential of dogs across transmission pathways, their involvement in ectoparasite-borne infections may be somewhat overrepresented relative to their confirmed biological competence. This trend could reflect a persistent symbolic association between dogs and external parasites such as fleas and ticks, shaped by their frequent infestation and reinforced in both scientific and public discourse. In the literature, dogs are on average cited more frequently in connection with tick- and flea-borne zoonoses than with other pathogen groups, despite being competent hosts for only a limited subset of these agents. By cross-referencing bibliometric data with empirical assessments of host competence, this integrative approach makes it possible to identify potential overestimations and gaps in current biological knowledge. It also helps to reveal how sociogeographic representations and taxonomic proximity may influence the visibility of certain host–pathogen associations, highlighting the importance of a critical perspective when interpreting bibliometric signals.

### 4.6. Evaluation and Relevance of the Bibliometric Approach

Our review demonstrates the value of bibliometric methods in identifying broad epidemiological patterns and host involvement in zoonotic transmission [115, 116]. The publication counts presented in this study primarily reflect the scientific discourse more than confirmed host–pathogen associations. However, targeted literature searches on the role of dogs in the transmission of each pathogen to humans have supported the validity of the results. The strong correlation between the “Dog”s part' index and the degree of zoonotic involvement attributed to dogs (Figure 2) suggests that bibliometric indicators closely mirror the epidemiological patterns described in specialized literature. When paired with a critical assessment of host competence, this dual-review approach helps to identify research gaps with clear implications for public health. Cross-referencing bibliometric data with empirical evidence reveals mismatches between scientific attention and actual epidemiological relevance. In some cases, dogs appear overrepresented in the literature without confirmed zoonotic competence—as illustrated by *Toxocara cati* or *Bartonella henselae*, which are primarily associated with cats but occasionally co-cited with dogs. Conversely, well-established associations between dogs and certain pathogens may be underrepresented in publication databases, such as for *Ancylostoma ceylanicum* or *Microsporum canis*, where additional transmission cycles exist despite a clearly documented canine role.

These discrepancies underscore the limitations of bibliometric approaches and the importance of integrating eco-epidemiological expertise to avoid interpretative biases. Despite these limitations, bibliometric tools remain valuable for analyzing how host species are represented in the scientific literature. They support the identification of dominant transmission pathways and help anticipate potential emergence risks. Integrating machine learning techniques, such as co-occurrence pattern detection or AI-based text mining, could improve the resolution of host–pathogen association mapping across large bibliographic datasets [117–119]. These tools may help identify overlooked associations or novel risks and refine our understanding of the direct and indirect roles dogs may play in disease transmission cycles. Although lexical counts cannot precisely quantify the infectious burden attributable to dogs, they provide a useful framework for classifying transmission pathways based on host association frequency. This method enables interspecies comparisons and helps prioritize health risks according to both ecological significance and visibility in scientific discourse.

## 5. Conclusion

This study provides a comprehensive assessment of the zoonotic role of *C. lupus familiaris*, revealing the diverse transmission pathways through which dogs contribute to disease emergence. By combining a structured pathogen inventory, bibliometric analysis, and epidemiological review, our study identifies key transmission dynamics, underscores research gaps, and informs targeted public health interventions. Our findings demonstrate that canine involvement in pathogen transmission to humans varies significantly depending on the transmission pathways and the socio-ecological contexts in which dogs evolve. They can act as primary, secondary, or occasional transmission hosts for nearly half of the recognized zoonotic agents. Among these pathogens, a great diversity of nematodes, cestodes, and rickettsiae stands out in our study, these taxonomic groups being more frequently associated with dogs in the literature than bacteria and viruses. These significant differences between taxa can be largely explained by the ecological characteristics of pathogens, as dogs are more frequently mentioned in relation to soil-contact transmission, tick, or flea-borne pathways, and bite inoculations.

To mitigate the zoonotic risks associated with different canine ecotypes, targeted public health interventions must be adapted to their specific epidemiological roles. Stray dogs, due to their exposure to wildlife reservoirs and contaminated environments, contribute significantly to the persistence of environmentally transmitted parasites [51, 120] and may act as entry points for emerging pathogens [43, 64]. Integrating them into genomic surveillance networks could enhance early detection of zoonotic threats, particularly in high-risk transmission zones such as squares and urban parks [121–123], livestock farming areas [124, 125], and wetland ecosystems [126]. To control their impact on public health, large-scale deworming campaigns should be implemented [86, 127], alongside waste management and sanitation improvements to reduce fecal–oral transmission and limit soil and water contamination [76, 128]. Conversely, companion dogs, while generally benefiting from better veterinary care, pose higher risks for direct-contact transmission of zoonotic pathogens through bites [129] and close contact [98]. Their prolonged and repeated interactions with humans in enclosed settings favor the transmission of directly communicable pathogens within households [18]. Routine veterinary monitoring of pets, including antimicrobial-resistant bacteria surveillance, and responsible pet ownership practices are essential to reduce these risks [68, 69, 130]. Moreover, their close cohabitation with their owners makes them ideal epidemiological sentinels [131–133]—targeted serological surveillance of pet dogs could provide valuable insights into human exposure risks and help detect pathogens with a high potential for zoonotic spillover, including viruses [50]. By tailoring health strategies to different canine ecotypes, zoonotic risks can be effectively controlled, reducing the global burden of dog-mediated zoonoses.

More broadly, the findings of this review indicate that the figure of the “zoonotic dog” constructed in scientific literature aligns most closely with the profile of stray dogs, suggesting a dominant perception of dogs as exogenous threats. This polarization of representations is not without consequence: it structures societal relationships with different categories of dogs, influences perceived dangerousness, and justifies often asymmetrical management policies. Yet, this binary vision—though operationally convenient—tends to obscure intermediate profiles whose epidemiological contributions may be equally significant. It also reflects deep-rooted cognitive and cultural biases that steer surveillance and public health efforts toward certain types of dogs at the expense of a more nuanced, context-sensitive understanding of zoonotic risks. These results thus call for a deconstruction of the homogeneous image of “the Dog” as a zoonotic host. They call for a recontextualization of ecotypic representations within their specific social, territorial, and biological settings, in order to refine prevention strategies and better capture the complexity of the canine epidemiological interface.

This review introduces the concept of a canine epidemiological gateway, emphasizing the role of dogs in facilitating the transmission of environmental pathogens to humans. Each dog ecotype—defined by its lifestyle, mobility, social integration, and access to veterinary care—contributes differently to the emergence and spread of zoonotic agents. A refined classification of these ecotypes, incorporating key behavioral and ecological traits, would improve our ability to identify their specific roles in pathogen transmission [46, 47, 134]. To support this effort, scientific publications should adopt a precise and consistent terminology that allows for clearer attribution of dog categories to distinct zoonotic pathways. A better understanding of these ecotype-specific dynamics is essential for improving zoonotic risk assessments and guiding targeted disease control strategies. For example, free-roaming owned dogs may serve as key epidemiological links between wild and domestic settings [135]. Their high mobility increases their exposure to contaminated environments, while regular human contact enhances their potential as direct transmission vectors [85]. These characteristics make them potential facilitators of pathogen adaptation and dissemination across multispecies networks, underscoring the need for further investigation [136].

Advancing this framework requires a combination of modeling and field-based approaches. Spatial models should be strengthened to identify geographic hotspots where dogs contribute to pathogen spillover [137–139], while GPS tracking can help quantify contact patterns between dogs, wildlife, livestock, and humans, thus informing intervention strategies [140, 141]. In parallel, behavioral and socioeconomic studies are needed to examine how hygiene practices, access to veterinary care, and human–dog relationships influence zoonotic transmission risks. Coupling these empirical approaches with bibliometric data could enhance the predictive value of literature-based tools and support more targeted health measures [142–144]. This integrated strategy helps bridge the gap between large-scale bibliometric evidence and operational zoonotic risk assessment. It supports the development of ecotype-specific interventions that reflect the diversity of epidemiological roles across dog populations. A coordinated, evidence-based approach—combining surveillance, prevention, and targeted responses—is critical to reduce the global burden of dog-mediated zoonoses. To this end, canine populations should be systematically included in One Health biosurveillance frameworks, leveraging their epidemiological significance to improve outbreak detection, prevent disease emergence, and inform control strategies at the human–animal–environment interface.

## Figures and Tables

**Table 1:** List of zoonotic pathogens transmissible by dogs.

Pathogen	Taxo group	Health impact	Exposure source	Pathway	Dog's part	Dog's partition	Zoonotic source potential
*Helicobacter canis*	Bacteria	Low	Animals	Close contact	0.882	0.252	Primary source
*Trichuris vulpis*	Nematoda	Low	Environment	Unsanitary conditions	0.827	−0.049	Primary source
*Ehrlichia canis*	Rickettsial	Moderate	Vectors	Ectoparasit vector	0.824	−0.116	Primary source
*Capnocytophaga canimorsus*	Bacteria	Moderate	Animals	Bites inoculation	0.715	−1.230	Primary source
*Staphylococcus pseudintermedius* MR	Bacteria	Moderate	Animals	Bites inoculation	0.714	−1.209	Primary source
*Brucella canis*	Bacteria	Moderate	Animals	Close contact	0.710	−0.280	Primary source
*Dipylidium caninum*	Cestoda	Low	Vectors	Accidental ingestion	0.709	0.314	Primary source
*Uncinaria stenocephala*	Nematoda	Moderate	Environment	Soil transmitted	0.685	−0.239	Primary source
*Bartonella vinsonii* berkhoffii	Bacteria	Low	Vectors	Bites inoculation	0.673	−0.025	Primary source
*Anaplasma platys*	Rickettsial	Low	Vectors	Ectoparasit vector	0.665	0.088	Primary source
*Dirofilaria repens*	Nematoda	Moderate	Vectors	Flying vector	0.629	−0.342	Primary source
*Dirofilaria immitis*	Nematoda	Moderate	Vectors	Flying vector	0.622	−0.248	Primary source
*Thelazia callipaeda*	Nematoda	Low	Vectors	Flying vector	0.607	−0.885	Primary source
*Ancylostoma braziliense*	Nematoda	Moderate	Environment	Soil transmitted	0.583	0.301	Primary source
*Cryptosporidium canis*	Protozoa	Moderate	Environment	Unsanitary conditions	0.582	−0.368	Primary source
*Streptococcus canis*	Bacteria	Low	Animals	Bites inoculation	0.551	−1.362	Primary source
*Ehrlichia ewingii*	Rickettsial	Moderate	Vectors	Ectoparasit vector	0.548	−0.477	Primary source
*Ancylostoma caninum*	Nematoda	Moderate	Environment	Soil transmitted	0.508	−0.054	Primary source
*Toxocara cati*	Nematoda	High	Environment	Unsanitary conditions	0.491	0.136	Occasional source
*Eucoleus aerophilus*	Nematoda	Low	Environment	Unsanitary conditions	0.482	−0.436	Primary source
*Toxocara canis*	Nematoda	High	Environment	Unsanitary conditions	0.423	−0.104	Primary source
*Chlamydia felis*	Bacteria	Low	Animals	Close contact	0.403	−0.357	Potential source
*Heterobilharzia americana*	Trematoda	Moderate	Environment	Soil transmitted	0.381	0.000	Primary source
*Leishmania infantum*	Protozoa	Priority	Vectors	Flying vector	0.380	−0.249	Primary source
*Leptospira interrogans* Canicola	Bacteria	High	Environment	Close contact	0.362	0.120	Primary source
*Rickettsia felis*	Rickettsial	Moderate	Vectors	Ectoparasit vector	0.342	−0.368	Secondary source
*Dracunculus medinensis*	Nematoda	High	Environment	Waterborne	0.333	0.301	Secondary source
*Ancylostoma ceylanicum*	Nematoda	Moderate	Environment	Soil transmitted	0.323	−0.058	Primary source
*Staphylococcus intermedius* MR	Bacteria	High	Animals	Close contact	0.319	−1.498	Primary source
*Neospora caninum*	Protozoa	Low	Food	Animal product	0.311	0.183	Primary source
*Campylobacter upsaliensis*	Bacteria	Moderate	Food	Animal product	0.308	−0.881	Primary source
*Taenia multiceps*	Cestoda	Moderate	Food	Animal product	0.302	0.058	Primary source
*Linguatula serrata*	Nematoda	Moderate	Food	Animal product	0.287	0.067	Primary source
*Anaplasma phagocytophilum*	Rickettsial	Moderate	Vectors	Ectoparasit vector	0.236	0.171	Secondary source
*Rickettsia rickettsii*	Rickettsial	High	Vectors	Ectoparasit vector	0.235	−0.512	Secondary source
*Leptospira interrogans* Icterohaemorrhagiae	Bacteria	Priority	Environment	Waterborne	0.234	0.131	Secondary source
*Angiostrongylus* spp.	Nematoda	Moderate	Food	Accidental ingestion	0.227	−0.559	Potential source
*Ehrlichia chaffeensis*	Rickettsial	Moderate	Vectors	Ectoparasit vector	0.226	−0.109	Secondary source
*Mesocestoides* spp.	Cestoda	Low	Food	Animal product	0.220	0.084	Primary source
*Echinococcus granulosus* (sl)	Cestoda	Priority	Food	Animal product	0.215	0.223	Secondary source
*Sarcocystis* spp.	Protozoa	Low	Food	Animal product	0.202	−0.301	Potential source
*Leishmania chagasi*	Protozoa	Priority	Vectors	Flying vector	0.189	0.176	Secondary source
*Malassezia* spp.	Fungi	Low	Animals	Close contact	0.180	−0.632	Secondary source
*Leptospira interrogans* Copenhageni	Bacteria	Priority	Environment	Waterborne	0.173	0.255	Secondary source
*Bartonella henselae*	Bacteria	Moderate	Vectors	Bites inoculation	0.172	−0.180	Secondary source
*Spirometra* spp.	Cestoda	Moderate	Food	Fish consumption	0.157	0.030	Secondary source
*Microsporum canis*	Fungi	Moderate	Animals	Close contact	0.142	−0.745	Primary source
Rabies virus	Virus	Priority	Animals	Bites inoculation	0.140	0.019	Primary source
*Bartonella* spp.	Bacteria	Moderate	Vectors	Ectoparasit vector	0.139	−0.186	Occasional source
*Corynebacterium ulcerans*	Bacteria	Low	Animals	Close contact	0.138	−0.903	Secondary source
*Bordetella bronchiseptica*	Bacteria	Low	Animals	Inhalation	0.132	−1.176	Secondary source
*Trichinella nativa*	Nematoda	Moderate	Food	Animal product	0.123	0.477	Occasional source
*Dibothriocephalus latus*	Cestoda	Moderate	Food	Fish consumption	0.122	0.222	Secondary source
*Echinococcus multilocularis*	Cestoda	Priority	Food	Animal product	0.120	−0.167	Secondary source
Canine Influenza	Virus	Low	Animals	Inhalation	0.112	−0.769	Primary source
*Trichinella britovi*	Nematoda	High	Food	Animal product	0.109	0.477	Potential source
*Anaplasma ovis*	Rickettsial	Low	Vectors	Ectoparasit vector	0.105	−0.125	Potential source
*Metagonimus* spp.	Trematoda	Moderate	Food	Fish consumption	0.100	0.000	Secondary source
*Baylisascaris* spp.	Nematoda	Moderate	Environment	Unsanitary conditions	0.097	−0.653	Occasional source
*Necator americanus*	Nematoda	High	Environment	Soil transmitted	0.094	−0.921	Potential source
*Brugia pahangi*	Nematoda	Low	Vectors	Flying vector	0.089	0.477	Secondary source
*Macracanthorhynchus* spp.	Acanthocephala	Low	Food	Animal product	0.085	0.222	Potential source
*Calodium hepaticum*	Nematoda	Low	Environment	Unsanitary conditions	0.083	−0.176	Occasional source
*Rickettsia typhi*	Rickettsial	Moderate	Vectors	Ectoparasit vector	0.081	−0.368	Potential source
*Borrelia burgdorferi* (sl)	Bacteria	Priority	Vectors	Ectoparasit vector	0.077	−0.377	Secondary source
*Alaria* spp.	Trematoda	Low	Food	Fish consumption	0.073	−0.439	Secondary source
*Paragonimus* spp.	Trematoda	High	Food	Fish consumption	0.072	0.000	Secondary source
*Opistorchis* spp.	Trematoda	High	Food	Fish consumption	0.070	0.301	Secondary source
*Rickettsia* spp.	Rickettsial	Moderate	Vectors	Ectoparasit vector	0.069	−0.164	Secondary source
*Leishmania braziliensis*	Protozoa	Priority	Vectors	Flying vector	0.069	0.000	Secondary source
*Giardia duodenalis*	Protozoa	Priority	Environment	Waterborne	0.067	−0.289	Secondary source
*Rickettsia conorii*	Rickettsial	Moderate	Vectors	Ectoparasit vector	0.065	−0.146	Secondary source
Barmah Forest virus	Virus	Low	Vectors	Flying vector	0.065	−0.602	Secondary source
*Taenia crassiceps*	Cestoda	Low	Food	Animal product	0.065	−0.398	Secondary source
*Toxoplasma gondii*	Protozoa	Priority	Food	Animal product	0.064	−0.072	Occasional source
*Strongyloides stercoralis*	Nematoda	High	Environment	Soil transmitted	0.063	−0.234	Secondary source
*Cryptosporidium* spp.	Protozoa	Moderate	Environment	Unsanitary conditions	0.060	−0.301	Occasional source
*Brucella suis*	Bacteria	Moderate	Animals	Close contact	0.060	−0.176	Occasional source
*Haplorchis* spp.	Trematoda	Moderate	Food	Fish consumption	0.058	0.000	Secondary source
*Gnathostoma spinigerum*	Nematoda	High	Food	Fish consumption	0.057	0.301	Secondary source
*Pasteurella multocida*	Bacteria	Moderate	Animals	Bites inoculation	0.056	−0.993	Secondary source
*Gnathostoma* spp.	Nematoda	High	Food	Fish consumption	0.056	0.301	Potential source
*Streptococcus equi* zooepidemicus	Bacteria	Moderate	Animals	Close contact	0.055	−0.477	Occasional source
*Leishmania mexicana*, tropica	Protozoa	Priority	Vectors	Flying vector	0.054	−0.092	Secondary source
*Yersinia pestis*	Bacteria	Priority	Vectors	Ectoparasit vector	0.053	−0.845	Secondary source
*Borrelia garinii*	Bacteria	Priority	Vectors	Ectoparasit vector	0.049	−0.602	Occasional source
Tick-borne relapsing fever *Borrelia*	Bacteria	Moderate	Vectors	Ectoparasit vector	0.045	−0.903	Occasional source
*Sporothrix brasiliensis*	Fungi	High	Animals	Bites inoculation	0.040	0.000	Occasional source
*Trichophyton mentagrophytes*	Fungi	Moderate	Animals	Close contact	0.040	−0.517	Secondary source
Louping-ill virus	Virus	Low	Vectors	Ectoparasit vector	0.040	−0.301	Potential source
*Coxiella burnetii*	Rickettsial	Priority	Animals	Inhalation	0.038	−0.412	Occasional source
*Blastocystis* spp.	Protozoa	Low	Environment	Unsanitary conditions	0.038	−0.103	Occasional source
*Sporothrix schenckii*	Fungi	Moderate	Animals	Soil transmitted	0.034	−0.602	Occasional source
*Hymenolepis nana*	Cestoda	Moderate	Food	Accidental ingestion	0.034	0.000	Occasional source
*Schistosoma mekongi*	Trematoda	High	Environment	Waterborne	0.033	0.000	Occasional source
Omsk hemorrhagic fever virus	Virus	High	Vectors	Ectoparasit vector	0.033	0.000	Potential source
*Angiostrongylus costaricensis*	Nematoda	Moderate	Food	Accidental ingestion	0.032	−0.477	Occasional source
*Trypanosoma cruzi*	Protozoa	Priority	Vectors	Ectoparasit vector	0.031	−0.336	Secondary source
Tick-borne encephalitis virus	Virus	Priority	Vectors	Ectoparasit vector	0.029	−0.903	Occasional source
*Balantidium coli*	Protozoa	Low	Environment	Unsanitary conditions	0.029	0.000	Occasional source
*Microsporum* spp.	Fungi	Low	Animals	Close contact	0.028	−0.368	Secondary source
*Cryptocotyle lingua*	Trematoda	Low	Food	Fish consumption	0.028	0.000	Occasional source
*Centrocestus* spp.	Trematoda	Low	Food	Fish consumption	0.028	0.000	Occasional source
*Opistorchis viverrini*	Trematoda	High	Food	Fish consumption	0.027	0.000	Secondary source
*Campylobacter coli*	Bacteria	Moderate	Food	Animal product	0.027	−0.632	Secondary source
*Erysipelothrix rhusiopathiae*	Bacteria	Moderate	Animals	Close contact	0.026	−0.477	Potential source
*Francisella tularensis*	Bacteria	Priority	Animals	Bites inoculation	0.025	−0.954	Occasional source
*Streptobacillus moniliformis*	Bacteria	Low	Animals	Bites inoculation	0.024	−0.477	Secondary source
*Clostridium perfringens*	Bacteria	High	Food	Animal product	0.024	−1.161	Occasional source
*Ascaris lumbricoides*	Nematoda	High	Environment	Unsanitary conditions	0.023	−0.505	Occasional source
EEE virus	Virus	Priority	Vectors	Flying vector	0.023	0.000	Potential source
Powassan virus	Virus	Moderate	Vectors	Ectoparasit vector	0.022	0.000	Potential source
*Brucella abortus*	Bacteria	Priority	Animals	Animal product	0.021	−0.125	Occasional source
*Trichinella spiralis*	Nematoda	High	Food	Animal product	0.021	0.477	Occasional source
*Brucella melitensis*	Bacteria	Priority	Animals	Animal product	0.021	−0.079	Occasional source
*Taenia solium*	Cestoda	Priority	Food	Animal product	0.021	0.000	Occasional source
*Arcobacter* spp.	Bacteria	Moderate	Food	Animal product	0.020	−0.813	Occasional source
*Cryptosporidium parvum*	Protozoa	Priority	Environment	Unsanitary conditions	0.019	−0.477	Occasional source
*Trichophyton* spp.	Fungi	Moderate	Animals	Close contact	0.017	−0.477	Occasional source
*Campylobacter jejuni*	Bacteria	Priority	Food	Animal product	0.017	−0.897	Secondary source
*Leishmania donovani* donovani	Protozoa	Priority	Vectors	Flying vector	0.016	−0.380	Potential source
*Mycoplasma* spp.	Bacteria	Low	Animals	Close contact	0.014	−0.253	Occasional source
*Moniliformis moniliformis*	Acanthocephala	Low	Food	Accidental ingestion	0.014	−0.176	Potential source
*Clostridioides difficile*	Bacteria	High	Environment	Unsanitary conditions	0.013	−1.447	Occasional source
*Acanthocephalus* spp.	Acanthocephala	Low	Food	Fish consumption	0.013	0.058	Potential source
*Schistosoma japonicum*	Trematoda	High	Environment	Waterborne	0.013	0.000	Occasional source
Murray Valley encephalitis virus	Virus	Moderate	Vectors	Flying vector	0.012	0.000	Occasional source
West Nile virus	Virus	Priority	Vectors	Flying vector	0.012	−0.681	Potential source
*Clonorchis sinensis*	Trematoda	High	Food	Fish consumption	0.011	−0.125	Occasional source
*Echinostoma* spp.	Trematoda	Moderate	Food	Fish consumption	0.011	0.176	Occasional source
Ross River virus	Virus	Moderate	Vectors	Flying vector	0.010	0.000	Potential source
*Campylobacter fetus*	Bacteria	Low	Food	Animal product	0.010	−0.301	Potential source
*Entamoeba histolytica*	Protozoa	High	Environment	Unsanitary conditions	0.009	−0.192	Occasional source
*Yersinia enterocolitica*	Bacteria	Moderate	Food	Animal product	0.009	−1.041	Occasional source
Japanese encephalitis virus	Virus	Priority	Vectors	Flying vector	0.007	−0.176	Potential source
Schistosome cercariae	Trematoda	Moderate	Environment	Waterborne	0.007	0.000	Occasional source
*Yersinia pseudotuberculosis*	Bacteria	Moderate	Food	Animal product	0.007	−0.602	Potential source
*Salmonella bongori*	Bacteria	Moderate	Food	Animal product	0.007	0.000	Potential source
Rotavirus	Virus	High	Environment	Unsanitary conditions	0.007	−0.301	Potential source
*Mycobacterium bovis*	Bacteria	Priority	Animals	Animal product	0.007	−0.845	Occasional source
*Staphylococcus aureus* MR	Bacteria	Priority	Animals	Close contact	0.006	−1.439	Secondary source
Hantavirus	Virus	Priority	Environment	Inhalation	0.006	−0.301	Potential source
Hepatitis E virus	Virus	High	Food	Animal product	0.006	−0.410	Occasional source
*Escherichia coli*	Bacteria	Priority	Environment	Unsanitary conditions	0.006	−1.010	Secondary source
Rift Valley fever virus	Virus	Priority	Vectors	Flying vector	0.005	−0.602	Occasional source
*Pseudomonas aeruginosa*	Bacteria	Moderate	Animals	Close contact	0.005	−0.916	Secondary source
*Schistosoma mansoni*	Trematoda	High	Environment	Waterborne	0.005	0.000	Potential source
*Mycobacterium ulcerans*	Bacteria	Moderate	Environment	Soil transmitted	0.004	0.000	Potential source
*Salmonella enterica*	Bacteria	Priority	Food	Animal product	0.004	−0.778	Secondary source
*Pneumocystis jirovecii*	Fungi	High	Animals	Inhalation	0.004	−0.301	Potential source
*Salmonella typhimurium*	Bacteria	High	Food	Animal product	0.004	−1.190	Potential source
*Listeria monocytogenes*	Bacteria	Priority	Food	Animal product	0.004	−0.970	Potential source
*Bacillus anthracis*	Bacteria	Priority	Environment	Soil transmitted	0.004	−0.301	Potential source
*Candida* spp.	Fungi	High	Animals	Close contact	0.003	−0.699	Potential source
*Vibrio cholerae*	Bacteria	High	Environment	Unsanitary conditions	0.002	0.000	Potential source
Lassa virus	Virus	Priority	Animals	Close contact	0.002	0.000	Potential source
*Aeromonas hydrophila*	Bacteria	Moderate	Environment	Waterborne	0.001	0.000	Potential source
*Burkholderia pseudomallei*	Bacteria	Priority	Environment	Soil transmitted	0.001	−0.301	Potential source
*Burkholderia mallei*	Bacteria	Priority	Animals	Close contact	0.000	0.000	Potential source

## Data Availability

All data used in this study are publicly available through the Web of Science Core Collection bibliographic database (Clarivate). The list of publications included in the bibliometric analysis is compiled and referenced in Table 1 of the manuscript.

## Supporting Information

Supporting Information 1 Table S1: List of zoonotic pathogens.

Supporting Information 2 χ^2^ adjusted standardized residuals. Probability graph of citing “dogs” and “pigs” on the total by transmission pathways. Summary, Results, Analysis of deviance, and Predicted probabilities of Model 1. Summary, Results, Analysis of deviance, and Predicted probabilities of Model 2. Summary, Results, Analysis of deviance, and Predicted probabilities of Model 3. Zoonotic pathogens list analysis code. Bibliometric analysis code.
