# Supplementary material for: Pet and Stray Dogs' Contribution to Zoonotic Transmission Pathways: A Bibliometric Review
Source: Transbound Emerg Dis. 2025 Aug 25;2025:5522451. doi: 10.1155/tbed/5522451 (PMC12401604; doi:10.1155/tbed/5522451)
Supplement: Supporting Information 2 — χ2 adjusted standardized residuals. Probability graph of citing “dogs” and “pigs” on the total by transmission pathways. Summary, Results, Analysis of deviance, and Predicted probabilities of Model 1. Summary, Results, Analysis of deviance, and Predicted probabilities of Model 2. Summary, Results, Analysis of deviance, and Predicted probabilities of Model 3. Zoonotic pathogens list analysis code. Bibliometric analysis code. [file 5522451.f2.docx]

# Supplementary materials

- **χ² adjusted standardized residuals**
- **Probability graph of citing ‘dogs’ and ‘pigs’ on the total by transmission pathways**
- **Summary, Results, Analysis of deviance, and Predicted probabilities of Model 1**
- **Summary, Results, Analysis of deviance, and Predicted probabilities of Model 2**
- **Summary, Results, Analysis of deviance, and Predicted probabilities of Model 3**
- **Zoonotic pathogens list analysis code**
- **Bibliometric analysis code**


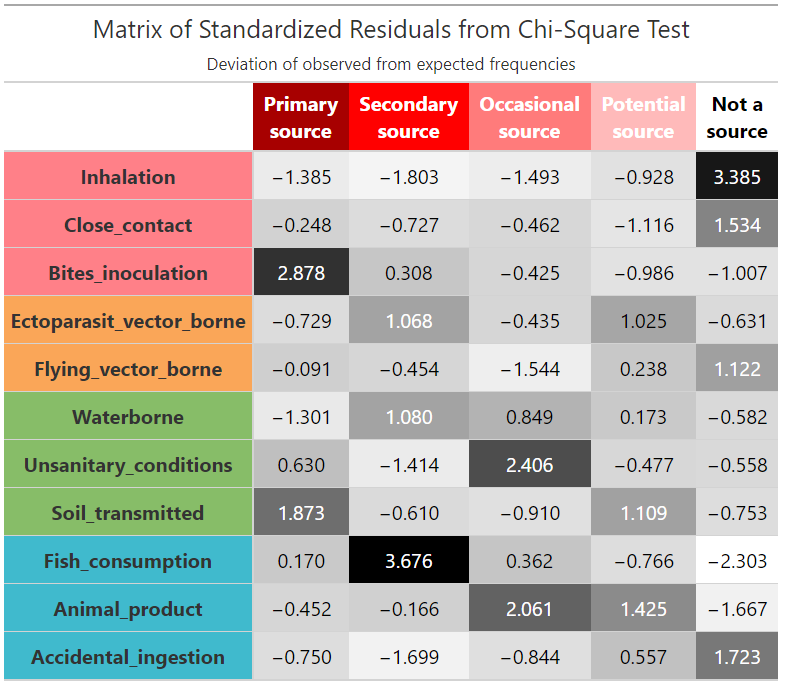


**Probability graph of citing ‘dogs’ and ‘pigs’ on the total by transmission pathways**

**
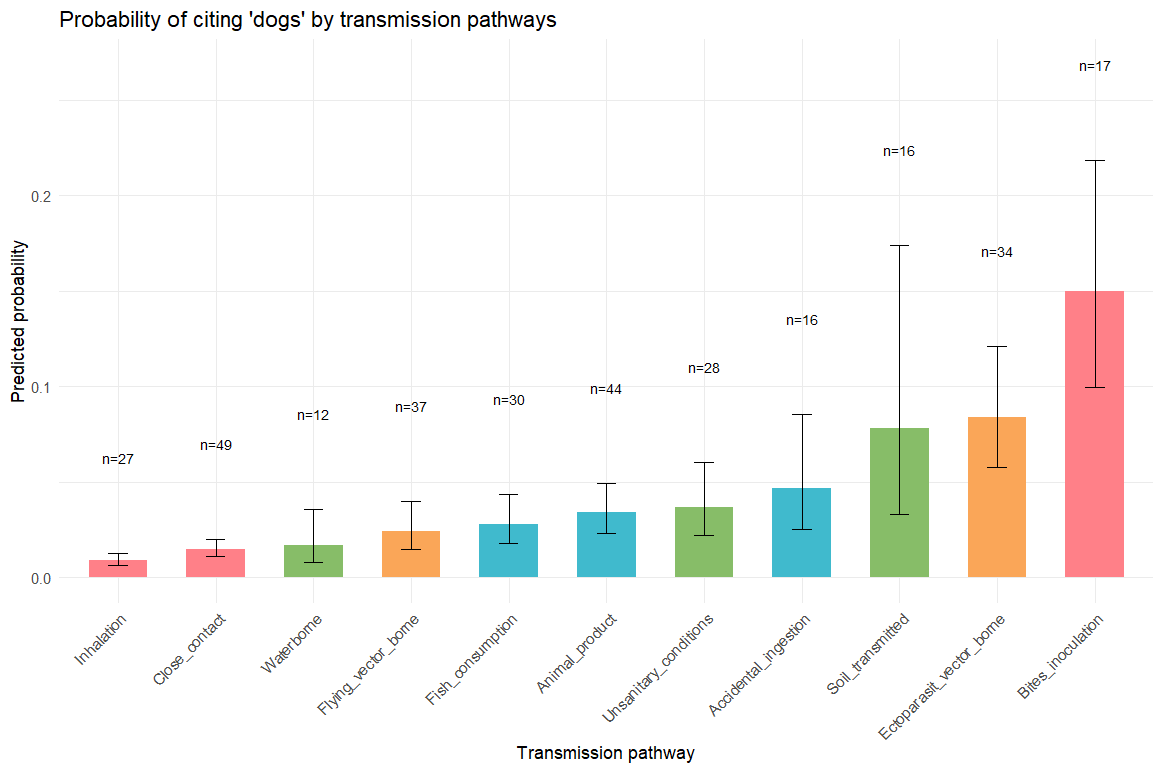
**

**
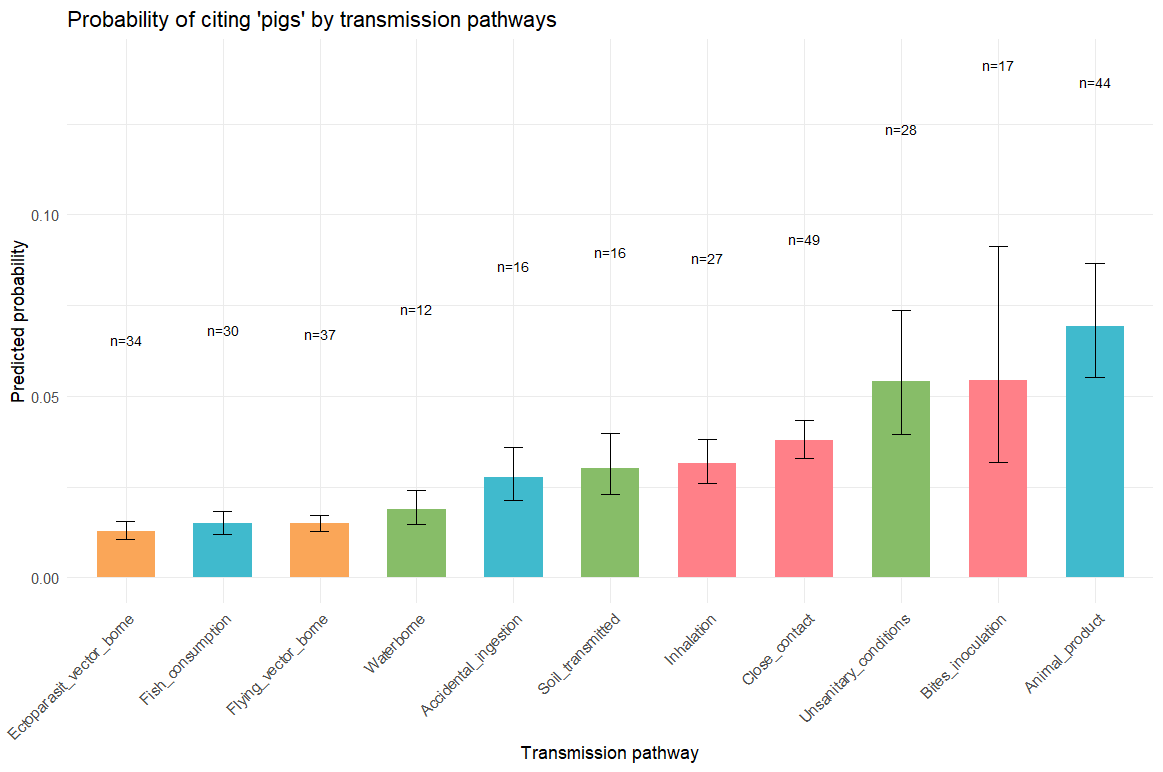
**

**Summary, Results, Analysis of deviance, and Predicted probabilities of Model 1**


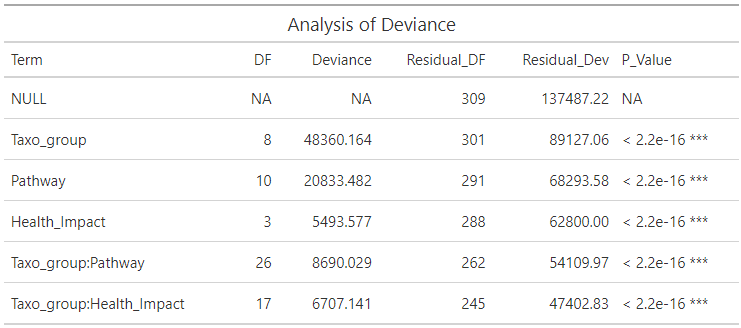
logit(p) = β0

+ β1​(Pathway)

+ β2​(Taxo_group)

+ β3​(Health_Impact)

+ β4(Pathway×Taxo_group)

+ β5(Health_Impact×Taxo_group)

**where p represents the probability of citing dogs over total**


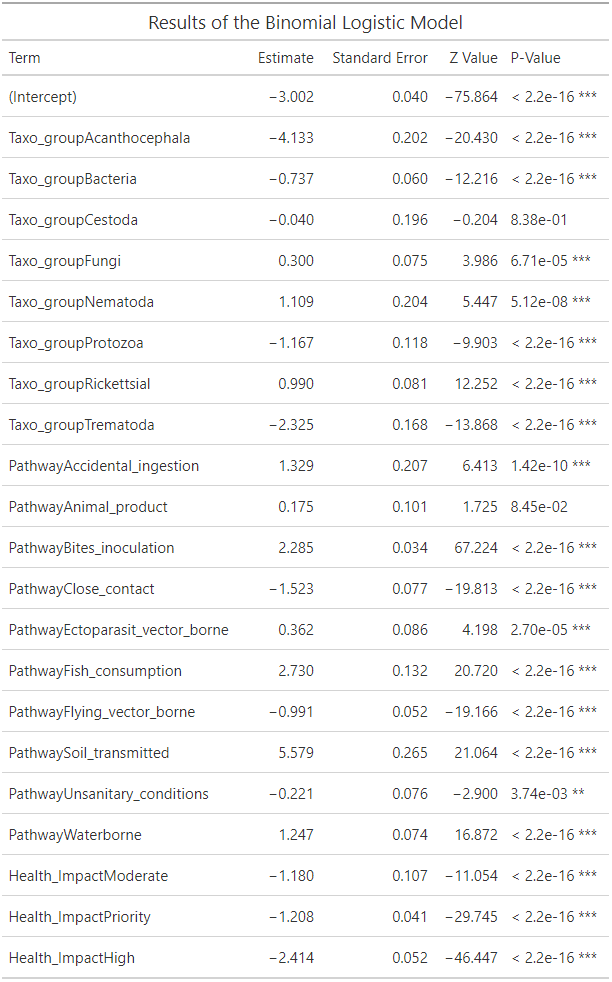

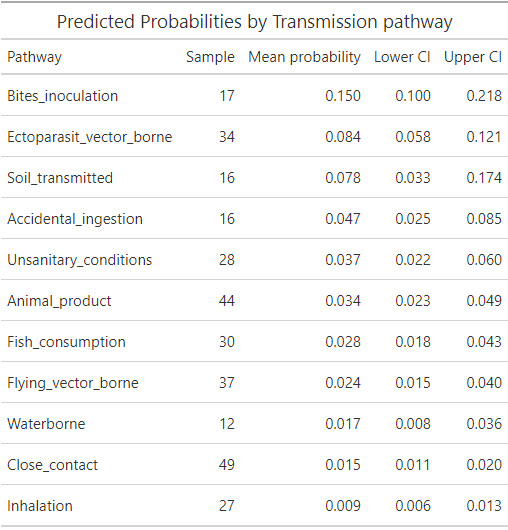

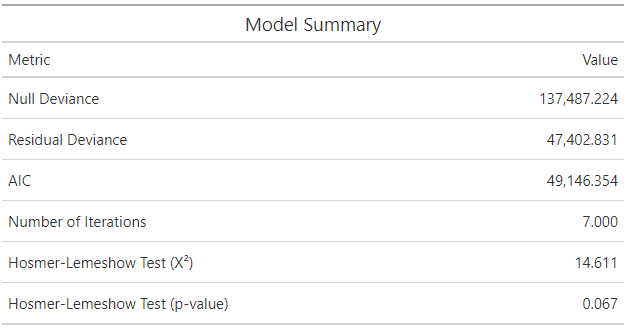


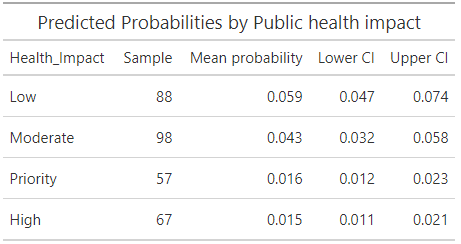

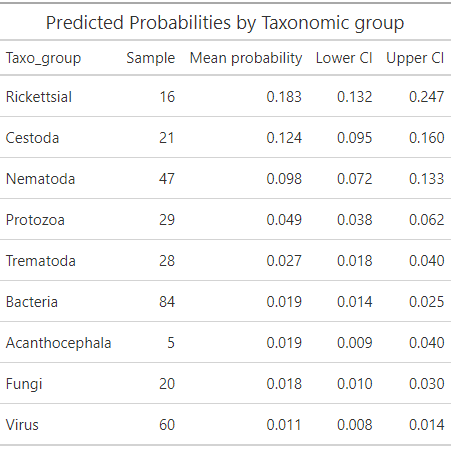


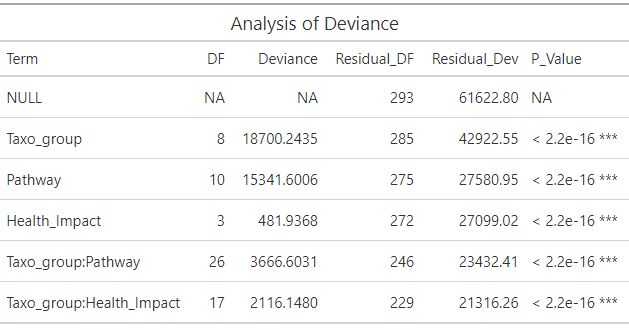
**Summary, Results, Analysis of deviance, and Predicted probabilities of Model 2**

logit(p) = β0

+ β1​(Pathway)

+ β2​(Taxo_group)

+ β3​(Health_Impact)

+ β4(Pathway×Taxo_group)

+ β5(Health_Impact×Taxo_group),

**where p represents the probability of citing dogs over pigs**


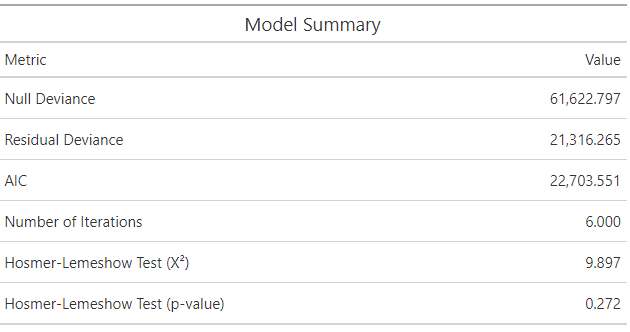

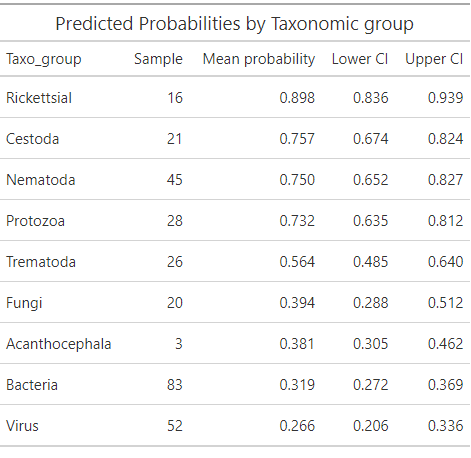

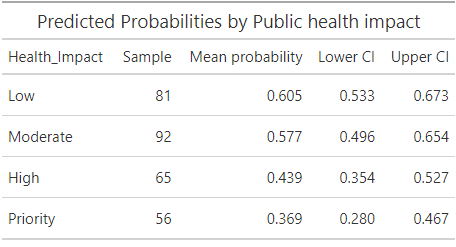

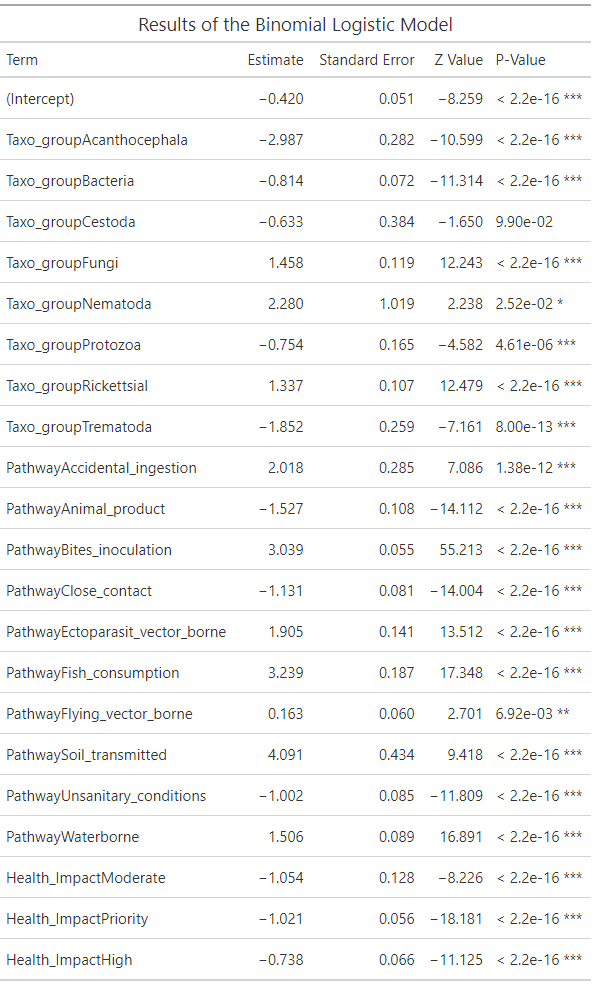

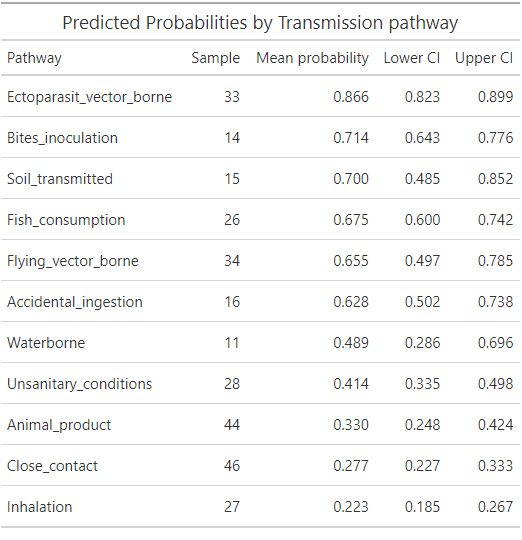


**Summary, Results, Analysis of deviance, and Predicted probabilities of Model 3**


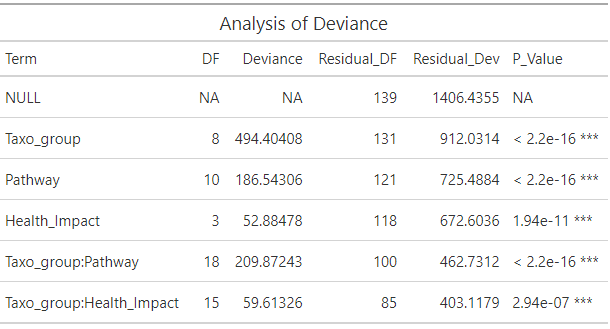
logit(p) = β0

+ β1​(Pathway)

+ β2​(Taxo_group)

+ β3​(Health_Impact)

+ β4(Pathway×Taxo_group)

+ β5(Health_Impact×Taxo_group),

**where p represents the probability of citing stray dogs over pet dogs**


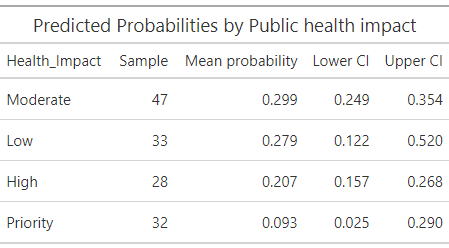

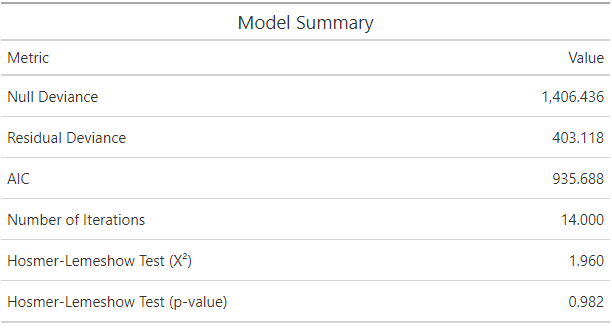

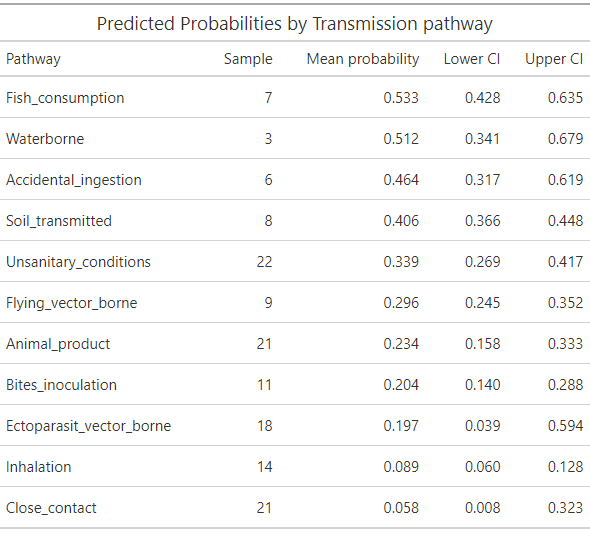

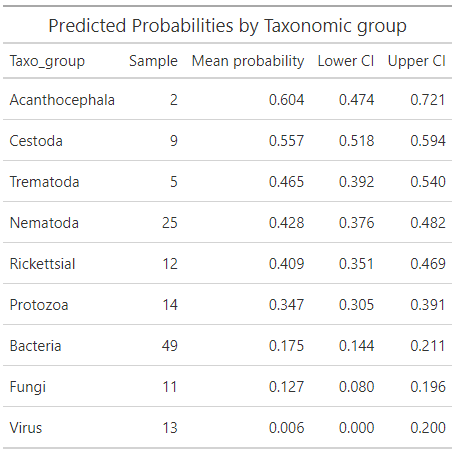

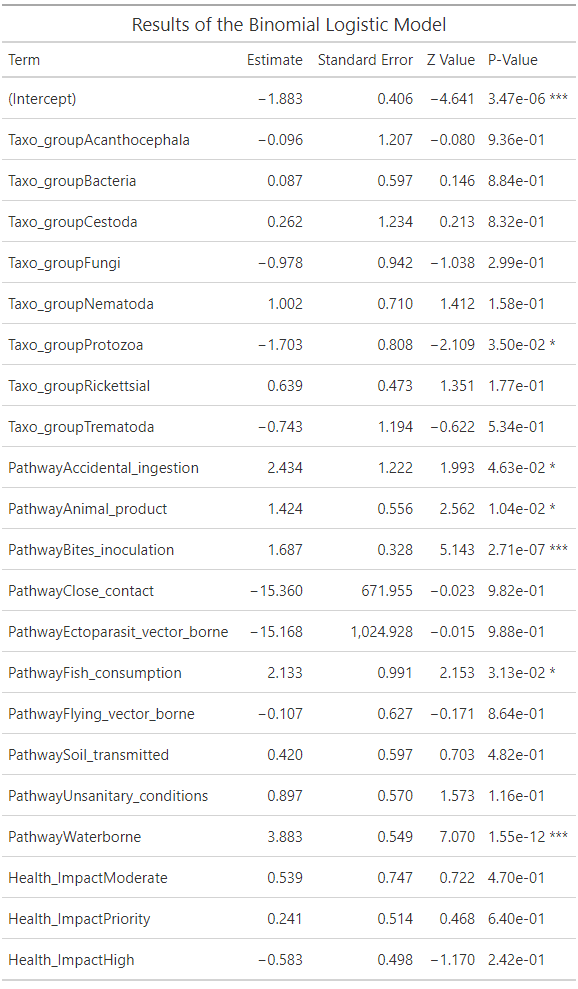


**Zoonotic pathogens list analysis code**

# libraries

library(dplyr)

library(tidyr)

library(ggplot2)

library(gt)

library(tibble)

# Importing the data

data <- read.csv("D:/Data/Zoonotic_table.csv", sep = ';', header = TRUE, stringsAsFactors = FALSE)

# Data preparation

prepare_data <- function(data) {

data <- data %>%

mutate(

across(c(Taxo_group, Exposure, Pathway, Dog_competency, Competency, Health_Impact, Zoonotic_source_potential), as.factor),

across(c(Total, With_dogs, With_stray_dogs, With_pet_dogs), as.numeric),

Ratio = (With_stray_dogs + 1) / (With_pet_dogs + 1),

Log_Ratio = log(Ratio)

) %>%

mutate(

Taxo_group = relevel(Taxo_group, ref = "Bacteria"),

Exposure = relevel(Exposure, ref = "Environment"),

Pathway = relevel(Pathway, ref = "Inhalation"),

Competency = relevel(Competency, ref = "NOT COMPETENT"),

Health_Impact = relevel(Health_Impact, ref = "Low")

)

return(data)

}

data$Zoonotic_source_potential <- factor(data$Zoonotic_source_potential,

levels = c("Primary source", "Secondary source", "Occasional source",

"Potential source", "Not a source"))

data$Pathway <- factor(data$Pathway, levels = c("Inhalation", "Close_contact","Bites_inoculation",

"Ectoparasit_vector_borne", "Flying_vector_borne",

"Waterborne", "Unsanitary_conditions","Soil_transmitted",

"Fish_consumption", "Animal_product", "Accidental_ingestion"))

data <- prepare_data(data)

data <- subset(data, (data$Competency== "COMPETENT" |data$Competency== "NOT COMPETENT"))

data$Competency <- droplevels(data$Competency)

data$Health_Impact <- droplevels(data$Health_Impact)

data$Zoonotic_source_potential <- droplevels(data$Zoonotic_source_potential)

# Define specific colors

color_mapping <- list(

"Exposure" = c("Animals" = "#FF8088", "Environment" = "#87BD68",

"Vectors" = "#FAA658", "Food" = "#40BACD"),

"Pathway" = c("Bites_inoculation" = "#FF8088", "Close_contact" = "#FF8088",

"Unsanitary_conditions" = "#87BD68", "Inhalation" = "#FF8088",

"Ectoparasit_vector_borne" = "#FAA658", "Flying_vector_borne" = "#FAA658",

"Accidental_ingestion" = "#40BACD", "Soil_transmitted" = "#87BD68",

"Animal_product" = "#40BACD", "Waterborne" = "#87BD68",

"Fish_consumption" = "#40BACD"),

"Taxo_group" = c("Bacteria" = "#FF69B4", "Virus" = "#FF4500", "Fungi" = "#8A2BE2",

"Rickettsial" = "#FF69B4", "Protozoa" = "#1E90FF", "Nematoda" = "#1E90FF",

"Trematoda" = "#1E90FF", "Cestoda" = "#1E90FF", "Acanthocephala" = '#1E90FF'),

"Health_Impact" = c("Low" = "#FFC0CB", "Moderate" = "#FF825E", "High" = "#FF4500",

"Priority" = "#8B0000"),

"Zoonotic_source_potential" = c("Primary source" = "#A70000", "Secondary source" = "#ff0000",

"Occasional source" = "#ff7b7b", "Potential source" = "#ffbaba", "Not a source" = "white")

)

# Créer un tableau de contingence

table_zoonotic <- table(data$Pathway, data$Zoonotic_source_potential)

chi2_zoonotic <- chisq.test(table_zoonotic)

residuals_matrix <- as.data.frame.matrix(chi2_zoonotic$residuals)

residuals_matrix <- residuals_matrix %>%

rownames_to_column(var = "Pathway")

# Extraire les informations du test de Chi²

chi2_results <- tibble(

"Statistic" = round(chi2_zoonotic$statistic, 3),

"Degrees of Freedom" = chi2_zoonotic$parameter,

"P-value" = formatC(chi2_zoonotic$p.value, format = "e", digits = 10),

"Significance" = ifelse(chi2_zoonotic$p.value < 0.05, "Significant (p < 0.05)", "Not Significant")

)

# Création du tableau gt avec formatage

chi2_results <- tibble(

"Chi² Statistic" = round(chi2_zoonotic$statistic, 3),

"Degrees of Freedom" = chi2_zoonotic$parameter,

"Exact P-value" = formatC(chi2_zoonotic$p.value, format = "e", digits = 10)

)

chi2_table <- chi2_results %>%

gt() %>%

tab_header(

title = "Chi-Square Test Results",

subtitle = "Summary of the Chi² test for independence"

) %>%

fmt_number(columns = "Chi² Statistic", decimals = 3) %>%

fmt_number(columns = "Degrees of Freedom", decimals = 0) %>%

cols_label(

`Chi² Statistic` = "Chi² Statistic",

`Degrees of Freedom` = "df",

`Exact P-value` = "Exact P-value"

) %>%

tab_style(

style = list(cell_text(weight = "bold")),

locations = cells_column_labels(everything())

)

chi2_table

# residuals table

table_gt_residuals_matrix <- residuals_matrix %>%

gt(rowname_col = "Pathway") %>%

tab_header(

title = "Matrix of Standardized Residuals from Chi-Square Test",

subtitle = "Deviation of observed from expected frequencies"

) %>%

fmt_number(columns = -Pathway, decimals = 3) %>%

data_color(

columns = -Pathway,

colors = scales::col_numeric(

palette = c("white", "grey", "black"), # Palette des résidus standardisés

domain = range(as.matrix(chi2_zoonotic$residuals), na.rm = TRUE)

)

) %>%

cols_align(align = "center") %>%

tab_style(

style = list(cell_text(weight = "bold")),

locations = cells_column_labels(everything())

)

for (source in names(color_mapping$Zoonotic_source_potential)) {

table_gt_residuals_matrix <- table_gt_residuals_matrix %>%

tab_style(

style = list(cell_fill(color = color_mapping$Zoonotic_source_potential[[source]]),

cell_text(weight = "bold", color = "white")),

locations = cells_column_labels(columns = source)

)

}

for (path in names(color_mapping$Pathway)) {

table_gt_residuals_matrix <- table_gt_residuals_matrix %>%

tab_style(

style = list(cell_fill(color = color_mapping$Pathway[[path]]),

cell_text(weight = "bold")),

locations = cells_stub(rows = Pathway == path) # CORRECTION : `cells_stub()`

)

}

table_gt_residuals_matrix <- table_gt_residuals_matrix %>%

tab_style(

style = list(cell_text(weight = "bold", color = "black")),

locations = cells_column_labels(columns = source)

)

table_gt_residuals_matrix

# Création d'un tableau de contingence

table_taxo <- table(data$Competency, data$Taxo_group)

print(table_taxo)

chi2_taxo <- chisq.test(table_taxo)

print(chi2_taxo)

print(chi2_taxo$residuals)

# Création d'un tableau de contingence

table_health <- table(data$Competency, data$Health_Impact)

print(table_health)

chi2_health <- chisq.test(table_health)

print(chi2_health)

print(chi2_health$residuals)

# Création d'un tableau de contingence

table_exposure <- table(data$Competency, data$Exposure)

print(table_exposure)

chi2_exposure <- chisq.test(table_exposure)

print(chi2_exposure)

print(chi2_exposure$residuals)

#################### Figure 1 ####################

# Filtrer les pathogènes COMPETENT en excluant Prion

data_long <- subset(data, (data$Taxo_group != "Prion")) %>%

count(Taxo_group, Zoonotic_source_potential) %>%

spread(key = Zoonotic_source_potential, value = n, fill = 0) %>%

gather(key = "Zoonotic_source_potential", value = "Count", -Taxo_group)

# Calculer les totaux et les proportions

data_long <- data_long %>%

group_by(Taxo_group) %>%

mutate(Total_pathway = sum(Count

Proportion = (Count / Total_pathway) * 100) %>%

ungroup()

# Créer un data frame pour les annotations `n=` au-dessus des barres

total_counts <- data_long %>%

group_by(Taxo_group) %>%

summarise(Total_n = sum(Count))

# Reclasser les facteurs pour garantir le bon affichage des catégories

data_long$Taxo_group <- factor(data_long$Taxo_group,

levels = c("Rickettsial", "Trematoda", "Cestoda", "Nematoda", "Bacteria",

"Acanthocephala", "Fungi", "Protozoa", "Virus",

"Not transmitted by dogs"))

data_long$Zoonotic_source_potential <- factor(data_long$Zoonotic_source_potential,

levels = c("Not a source", "Potential source", "Occasional source", "Secondary source", "Primary source"))

# Créer le graphique avec proportions et annotations

ggplot(data_long, aes(x = Taxo_group, y = Proportion, fill = Zoonotic_source_potential)) +

geom_bar(stat = "identity", position = "stack") +

scale_fill_manual(values = color_mapping$Zoonotic_source_potential) +

labs(title = "Proportion of zoonotic pathogens by taxonomic group",

x = "",

y = "Proportion of pathogens by zoonotic potential of dogs (%)",

fill = "Dogs as") +

theme_minimal() +

theme(axis.text.x = element_text(angle = 45, hjust = 1, size = 12),

text = element_text(size = 14),

plot.title = element_text(size = 14),

legend.position = "bottom") + # Légende en bas

geom_text(data = total_counts, aes(x = Taxo_group, y = 105, label = paste0("n=", Total_n)),

inherit.aes = FALSE,

color = "black",

size = 5)

#################### Figure 2 ####################

# Filtrer et compter les pathogènes COMPETENT par Pathway

data_competent <- subset(data, data$Competency == "COMPETENT") %>%

count(Pathway) %>%

rename(Count = n) %>%

mutate(Competency = "COMPETENT", Pathway_color = Pathway)

# Filtrer et compter les pathogènes NOT COMPETENT par Pathway

data_not_competent <- subset(data, data$Competency == "NOT COMPETENT") %>%

count(Pathway) %>%

rename(Count = n) %>%

mutate(Competency = "NOT COMPETENT", Pathway_color = "Not transmitted by dogs") # Coloration en noir

# Fusionner les deux jeux de données

data_pathway <- bind_rows(data_competent, data_not_competent)

# Définir l'ordre des Pathway en fonction du graphique fourni

data_pathway$Pathway <- factor(data_pathway$Pathway,

levels = c("Fish_consumption", "Bites_inoculation", "Waterborne",

"Animal_product", "Soil_transmitted", "Unsanitary_conditions",

"Ectoparasit_vector_borne", "Flying_vector_borne", "Close_contact",

"Accidental_ingestion", "Inhalation"))

# Convertir Competency en facteur pour contrôler l'ordre d'affichage

data_pathway$Competency <- factor(data_pathway$Competency,

levels = c("NOT COMPETENT", "COMPETENT"))

# Réorganisation du facteur Pathway_color en mettant "Black" en dernier

data_pathway$Pathway_color <- factor(data_pathway$Pathway_color,

levels = c("Not transmitted by dogs", "Ectoparasit_vector_borne", "Flying_vector_borne", "Animal_product", "Fish_consumption", "Accidental_ingestion", "Bites_inoculation", "Close_contact", "Inhalation", "Unsanitary_conditions", "Soil_transmitted", "Waterborne"))

# Calcul des proportions et total des pathogènes par Pathway

data_pathway <- data_pathway %>%

group_by(Pathway) %>%

mutate(Total_pathway = sum(Count),

Proportion = (Count / Total_pathway) * 100) %>%

ungroup()

# Créer un data frame distinct pour les annotations `n=`

total_counts <- data_pathway %>%

distinct(Pathway, Total_pathway) # Évite la répétition de valeurs

# Création du graphique avec proportions et annotations `n=` au-dessus

ggplot(data_pathway, aes(x = Pathway, y = Proportion, fill = Pathway_color)) +

geom_bar(stat = "identity", position = "stack") +

scale_fill_manual(values = c("Not transmitted by dogs" = "white", color_mapping$Pathway)) +

scale_x_discrete(labels = function(x) gsub("_", " ", x)) +

ylim(0, 110) +

labs(title = "Proportion of zoonotic pathogens by transmission pathways",

x = "",

y = "Proportion of pathogens transmitted by dogs (%)",

fill = "") +

theme_minimal() +

theme(axis.text.x = element_text(angle = 45, hjust = 1, size = 12),

text = element_text(size = 12),

plot.title = element_text(size = 14),

legend.position = "none") +

geom_text(data = total_counts, aes(x = Pathway, y = 105, label = paste0("n=", Total_pathway)),

inherit.aes = FALSE,

color = "black",

size = 5)

**Bibiliometric analysis code**

# libraries

library(emmeans)

library(dplyr)

library(ggplot2)

library(ggdendro)

library(gt)

# Importing the data

data <- read.csv("D:/Data /Zoonotic_table.csv",

sep = ';', header = TRUE, stringsAsFactors = FALSE)

# Data preparation

prepare_data <- function(data) {

data <- data %>%

mutate(

across(c(Taxo_group, Exposure, Pathway, Dog_competency, Health_Impact, Zoonotic_source_potential), as.factor),

across(c(Total, With_dogs, With_pigs, With_stray_dogs, With_pet_dogs), as.numeric),

Ratio = (With_stray_dogs + 1) / (With_pet_dogs + 1),

Log_Ratio = log(Ratio),

Ratio_pig = (With_dogs + 1) / (With_pigs + 1),

Log_Ratio_pig = log(Ratio_pig)

) %>%

filter(Competency %in% c("COMPETENT", "NOT COMPETENT")) %>%

mutate(

Taxo_group = relevel(Taxo_group, ref = "Virus"),

Exposure = relevel(Exposure, ref = "Animals"),

Pathway = relevel(Pathway, ref = "Inhalation"),

Health_Impact = relevel(Health_Impact, ref = "Low"),

)

return(data)

}

data <- prepare_data(data)

data$Zoonotic_source_potential <- factor(data$Zoonotic_source_potential,

levels = c("Not a source", "Potential source", "Rare source", "Second source", "First source"))

data$Health_Impact <- factor(data$Health_Impact,

levels = c("Low", "Moderate", "Priority", "High"))

data <- data %>%

mutate(Zoonotic_source_weight = as.numeric(Zoonotic_source_potential) / max(as.numeric(Zoonotic_source_potential), na.rm = TRUE))

data50 <- data %>% filter(Total > 49)

data50 <- subset(data50, data50$Taxo_group!="Prion")

data50 <- data50 %>%

filter(!is.na(With_dogs) & !is.na(With_pigs) &!is.na(Total) & !is.na(Pathway) & !is.na(Taxo_group) & !is.na(Health_Impact)) %>%

mutate(

Pathway = as.factor(Pathway),

Taxo_group = as.factor(Taxo_group),

Health_Impact = as.factor(Health_Impact)

)

data_pig <- data50 %>% filter((With_dogs + With_pigs) > 4)

data5 <- data %>% filter((With_pet_dogs + With_stray_dogs) > 4)

data5 <- subset(data5, data5$Taxo_group!="Prion")

data5 <- data5 %>%

filter(!is.na(With_dogs) & !is.na(With_pigs) & !is.na(Total) & !is.na(Pathway) & !is.na(Taxo_group) & !is.na(Health_Impact)) %>%

mutate(

Pathway = as.factor(Pathway),

Taxo_group = as.factor(Taxo_group),

Health_Impact = as.factor(Health_Impact)

)

# Define specific colors

color_mapping <- list(

"Exposure" = c("Animals" = "#FF8088", "Environment" = "#87BD68",

"Vectors" = "#FAA658", "Food" = "#40BACD"),

"Pathway" = c("Bites_inoculation" = "#FF8088", "Close_contact" = "#FF8088",

"Unsanitary_conditions" = "#87BD68", "Inhalation" = "#FF8088",

"Ectoparasit_vector_borne" = "#FAA658", "Flying_vector_borne" = "#FAA658",

"Accidental_ingestion" = "#40BACD", "Soil_transmitted" = "#87BD68",

"Animal_product" = "#40BACD", "Waterborne" = "#87BD68",

"Fish_consumption" = "#40BACD"),

"Taxo_group" = c("Bacteria" = "#FF69B4", "Virus" = "#FF4500", "Fungi" = "#8A2BE2",

"Rickettsial" = "#FF69B4", "Protozoa" = "#1E90FF", "Nematoda" = "#1E90FF",

"Trematoda" = "#1E90FF", "Cestoda" = "#1E90FF", "Acanthocephala" = '#1E90FF'),

"Zoonotic" = c("Not a source" = "white", "Potential source"= "#FFC0CB", "Rare source" = "#FF825E", "Second source" = "#FF4500", "First source" = "#8B0000"),

"Health_Impact" = c("Low" = "#FFC0CB", "Moderate" = "#FF825E", "High" = "#FF4500",

"Priority" = "#8B0000")

)

# Function to build a model and create a graph based on observation-level predictions

# Fonction principale

create_graph <- function(data, formula, color_category, title, x_label, y_label, category_prefix) {

model <- glm(formula, family = binomial, data = data)

# Extraction des métriques du modèle

model_summary <- tibble(

Metric = c("Null Deviance", "Residual Deviance", "AIC", "Number of Iterations"),

Value = c(model$null.deviance, model$deviance, AIC(model), model$iter)

)

# Test de Hosmer-Lemeshow

hoslem_test <- hoslem.test(model$y, fitted(model), g = 10)

model_summary <- model_summary %>%

add_row(Metric = "Hosmer-Lemeshow Test (X²)", Value = hoslem_test$statistic) %>%

add_row(Metric = "Hosmer-Lemeshow Test (p-value)", Value = hoslem_test$p.value)

# Table d'analyse de la variance

anova_table <- anova(model, test = "Chisq") %>%

as.data.frame() %>%

tibble::rownames_to_column("Term") %>%

rename(DF = Df, Deviance = Deviance, Residual_DF = `Resid. Df`, Residual_Dev = `Resid. Dev`, P_Value = `Pr(>Chi)`)

# Prédictions et intervalles de confiance

predicted_logit <- predict(model, type = "link", se.fit = TRUE)

data$Logit_Pred <- predicted_logit$fit

data$SE_Logit <- predicted_logit$se.fit

data$Predicted_Prob <- plogis(data$Logit_Pred)

coef_df <- data %>%

group_by_at(category_prefix) %>%

summarise(

n_obs = n(),

Mean_Logit = mean(Logit_Pred, na.rm = TRUE),

SE_Logit = ifelse(n_obs > 1, sd(Logit_Pred, na.rm = TRUE) / sqrt(n_obs), NA),

Lower_CI_Logit = Mean_Logit - 1.96 * SE_Logit,

Upper_CI_Logit = Mean_Logit + 1.96 * SE_Logit

) %>%

ungroup() %>%

mutate(

Mean_Prob = plogis(Mean_Logit),

Lower_CI = pmax(0, plogis(Lower_CI_Logit)),

Upper_CI = pmin(1, plogis(Upper_CI_Logit)),

Bar_Width = sqrt(n_obs) / max(sqrt(n_obs))

) %>%

arrange(desc(Mean_Prob))

# Résultats de la régression logistique

model_results <- broom::tidy(model) %>%

dplyr::select(term, estimate, std.error, statistic, p.value) %>%

rename(Term = term, Estimate = estimate, SE = std.error, Z_Value = statistic, P_Value = p.value)

# Affichage des tableaux avec `gt`

print(gt(model_summary) %>%

tab_header(title = "Model Summary") %>%

fmt_number(columns = c(Value), decimals = 3) %>%

tab_options(

table.font.size = pct(75),

table.width = pct(75)

)

)

print(

gt(anova_table %>%

mutate(

P_Value = case_when(

P_Value < 2.2e-16 ~ paste0("< 2.2e-16 ***"),

P_Value < 0.001 ~ paste0(formatC(P_Value, format = "e", digits = 2), " ***"),

P_Value < 0.01 ~ paste0(formatC(P_Value, format = "e", digits = 2), " **"),

P_Value < 0.05 ~ paste0(formatC(P_Value, format = "e", digits = 2), " *"),

P_Value > 0.05 ~ paste0(formatC(P_Value, format = "e", digits = 2)),

TRUE ~ as.character(P_Value)

)

)

) %>%

tab_header(title = "Analysis of Deviance") %>%

tab_options(

table.font.size = pct(75),

table.width = pct(75)

)

)

# Création du tableau gt

print(

gt(coef_df %>% dplyr::select(1, 2, 7, 8, 9)) %>%

tab_header(title = paste("Predicted Probabilities by", x_label)) %>%

cols_label(

n_obs = "Sample",

Mean_Prob = "Mean probability",

Lower_CI = "Lower CI",

Upper_CI = "Upper CI"

) %>%

fmt_number(columns = c(Mean_Prob, Lower_CI, Upper_CI), decimals = 3) %>%

cols_align(

align = "left",

columns = 1

) %>%

tab_options(

table.font.size = pct(75),

table.width = pct(75),

)

)

# Ajouter les étoiles directement dans la colonne P_Value

model_results <- model_results %>%

filter(!str_detect(Term, ":")) %>% # Supprimer les interactions

mutate(

P_Value = case_when(

P_Value < 2.2e-16 ~ paste0("< 2.2e-16 ***"),

P_Value < 0.001 ~ paste0(formatC(P_Value, format = "e", digits = 2), " ***"),

P_Value < 0.01 ~ paste0(formatC(P_Value, format = "e", digits = 2), " **"),

P_Value < 0.05 ~ paste0(formatC(P_Value, format = "e", digits = 2), " *"),

P_Value > 0.05 ~ paste0(formatC(P_Value, format = "e", digits = 2)),

TRUE ~ as.character(P_Value)

)

)

# Création du tableau gt

print(

gt(model_results) %>%

tab_header(title = "Results of the Binomial Logistic Model") %>%

cols_label(

Term = "Term",

Estimate = "Estimate",

SE = "Standard Error",

Z_Value = "Z Value",

P_Value = "P-Value"

) %>%

# Formater les nombres à 3 décimales

fmt_number(columns = c(Estimate, SE, Z_Value), decimals = 3) %>%

cols_align(

align = "left",

columns = "Term"

) %>%

# Réduire la taille du tableau et la hauteur des lignes

tab_options(

table.font.size = pct(75),

table.width = pct(75),

)

)

# Création du graphique avec largeur des barres proportionnelle à n_obs

ggplot(coef_df, aes(x = reorder(!!sym(category_prefix), Mean_Prob), y = Mean_Prob, fill = !!sym(category_prefix))) +

geom_col(width = 0.6, show.legend = FALSE) + # Largeur fixe pour toutes les barres

geom_errorbar(aes(ymin = Lower_CI, ymax = Upper_CI), width = 0.2) +

geom_text(aes(label = paste0("n=", n_obs), y = Upper_CI + 0.05),

color = "black", size = 3) +

scale_fill_manual(values = color_mapping[[color_category]], guide = "none") +

theme_minimal() +

labs(title = title, x = x_label, y = y_label) +

theme(axis.text.x = element_text(angle = 45, hjust = 1))

}

# Generate and display graphs successively

graphs <- list(

#list(data = data50, formula = cbind(With_dogs, Total - With_dogs) ~ Taxo_group * Pathway * Health_Impact - Pathway:Health_Impact - Pathway:Taxo_group:Health_Impact,

#color = "Health_Impact", title = "Probability of citing 'dogs' by Public health impact",

#x_label = "Public health impact", y_label = "Predicted probabiliy", category_prefix = "Health_Impact"),

list(data = data50, formula = cbind(With_pigs, Total - With_pigs) ~ Taxo_group * Pathway * Health_Impact - Pathway:Health_Impact - Pathway:Taxo_group:Health_Impact,

color = "Health_Impact", title = "Probability of citing 'pigs' by Public health impact",

x_label = "Public health impact", y_label = "Predicted probability", category_prefix = "Health_Impact"),

list(data = data_pig, formula = cbind(With_dogs, With_pigs) ~ Taxo_group * Pathway * Health_Impact - Pathway:Health_Impact - Pathway:Taxo_group:Health_Impact,

color = "Health_Impact", title = "Probability of citing 'dogs' vs. 'pigs' by Public health impact",

x_label = "Public health impact", y_label = "Predicted probability", category_prefix = "Health_Impact"),

list(data = data5, formula = cbind(With_stray_dogs, With_pet_dogs) ~ Taxo_group * Pathway * Health_Impact - Pathway:Health_Impact - Pathway:Taxo_group:Health_Impact,

color = "Health_Impact", title = "Probability of citing 'stray dogs' vs. 'pet dogs' by Public health impact",

x_label = "Public health impact", y_label = "Predicted probability", category_prefix = "Health_Impact"),

list(data = data50, formula = cbind(With_dogs, Total - With_dogs) ~ Taxo_group * Pathway * Health_Impact - Pathway:Health_Impact - Pathway:Taxo_group:Health_Impact,

color = "Taxo_group", title = "Probability of citing 'dogs' by taxonomic groups",

x_label = "Taxonomic group", y_label = "Predicted probability", category_prefix = "Taxo_group"),

list(data = data50, formula = cbind(With_pigs, Total - With_pigs) ~ Taxo_group * Pathway * Health_Impact - Pathway:Health_Impact - Pathway:Taxo_group:Health_Impact,

color = "Taxo_group", title = "Probability of citing 'pigs' by taxonomic groups",

x_label = "Taxonomic group", y_label = "Predicted probability", category_prefix = "Taxo_group"),

list(data = data_pig, formula = cbind(With_dogs, With_pigs) ~ Taxo_group * Pathway * Health_Impact - Pathway:Health_Impact - Pathway:Taxo_group:Health_Impact,

color = "Taxo_group", title = "Probability of citing 'dogs' vs. 'pigs' by taxonomic groups",

x_label = "Taxonomic group", y_label = "Predicted probability", category_prefix = "Taxo_group"),

list(data = data5, formula = cbind(With_stray_dogs, With_pet_dogs) ~ Taxo_group * Pathway * Health_Impact - Pathway:Health_Impact - Pathway:Taxo_group:Health_Impact,

color = "Taxo_group", title = "Probability of citing 'stray dogs' vs. 'pet dogs' by taxonomic groups",

x_label = "Taxonomic group", y_label = "Predicted probability", category_prefix = "Taxo_group"),

list(data = data50, formula = cbind(With_dogs, Total - With_dogs) ~ Taxo_group * Exposure * Health_Impact - Pathway:Health_Impact - Pathway:Taxo_group:Health_Impact,

color = "Exposure", title = "Probability of citing 'dogs' by sources of exposure",

x_label = "Source of exposure", y_label = "Predicted probability", category_prefix = "Exposure"),

list(data = data50, formula = cbind(With_pigs, Total - With_pigs) ~ Taxo_group * Exposure * Health_Impact - Pathway:Health_Impact - Pathway:Taxo_group:Health_Impact,

color = "Exposure", title = "Probability of citing 'pigs' by sources of exposure",

x_label = "Source of exposure", y_label = "Predicted probability", category_prefix = "Exposure"),

list(data = data_pig, formula = cbind(With_dogs, With_pigs) ~ Taxo_group * Exposure * Health_Impact - Pathway:Health_Impact - Pathway:Taxo_group:Health_Impact,

color = "Exposure", title = "Probability of citing 'dogs' vs. 'pigs' by sources of exposure",

x_label = "Source of exposure", y_label = "Predicted probability", category_prefix = "Exposure"),

list(data = data5, formula = cbind(With_stray_dogs, With_pet_dogs) ~ Taxo_group * Exposure * Health_Impact - Pathway:Health_Impact - Pathway:Taxo_group:Health_Impact,

color = "Exposure", title = "Probability of citing 'stray dogs' vs. 'pet dogs' by sources of exposure",

x_label = "Source of exposure", y_label = "Predicted probability", category_prefix = "Exposure"),

list(data = data50, formula = cbind(With_dogs, Total - With_dogs) ~ Taxo_group * Pathway * Health_Impact - Pathway:Health_Impact - Pathway:Taxo_group:Health_Impact,

color = "Pathway", title = "Probability of citing 'dogs' by transmission pathways",

x_label = "Transmission pathway", y_label = "Predicted probability", category_prefix = "Pathway"),

list(data = data50, formula = cbind(With_pigs, Total - With_pigs) ~ Taxo_group * Pathway * Health_Impact - Pathway:Health_Impact - Pathway:Taxo_group:Health_Impact,

color = "Pathway", title = "Probability of citing 'pigs' by transmission pathways",

x_label = "Transmission pathway", y_label = "Predicted probability", category_prefix = "Pathway"),

list(data = data_pig, formula = cbind(With_dogs, With_pigs) ~ Taxo_group * Pathway * Health_Impact - Pathway:Health_Impact - Pathway:Taxo_group:Health_Impact,

color = "Pathway", title = "Probability of citing 'dogs' vs. 'pigs' by transmission pathways",

x_label = "Transmission pathway", y_label = "Predicted probability", category_prefix = "Pathway"),

list(data = data5, formula = cbind(With_stray_dogs, With_pet_dogs) ~ Taxo_group * Pathway * Health_Impact - Pathway:Health_Impact - Pathway:Taxo_group:Health_Impact,

color = "Pathway", title = "Probability of citing 'stray dogs' vs. 'pet dogs' by transmission pathways",

x_label = "Transmission pathway", y_label = "Predicted probability", category_prefix = "Pathway"),

list(data = data50, formula = cbind(With_dogs, Total - With_dogs) ~ Zoonotic_source_potential * Pathway * Taxo_group,

color = "Zoonotic", title = "Probability of citing 'dogs' by zoonotic potentials",

x_label = "Zoonotic potential", y_label = "Predicted probability", category_prefix = "Zoonotic_source_potential"),

list(data = data5, formula = cbind(With_stray_dogs, With_pet_dogs) ~ Zoonotic_source_potential * Pathway * Taxo_group,

color = "Zoonotic", title = "Probability of citing 'stray dogs' vs. 'pet dogs' by zoonotic potentials",

x_label = "Zoonotic potential", y_label = "Predicted probability", category_prefix = "Zoonotic_source_potential")

)

for (graph in graphs) {

print(create_graph(graph$data, graph$formula, graph$color, graph$title, graph$x_label, graph$y_label, graph$category_prefix))

}
